# Hydrochemical and health risk evaluation of the groundwater in Gonbad Kavus area, northeastern Iran

**DOI:** 10.1038/s41598-026-51636-2

**Published:** 2026-05-06

**Authors:** Hasan Eteraf, Viktória Mikita, Balázs Kovács

**Affiliations:** Institute of Water and Environmental Management, Faculty of Earth Science, University of Miskolc, Miskolc, 3515 Hungary

**Keywords:** Groundwater Quality, Hydrogeochemistry, Health Risk Assessment, Multivariate Statistical Analysis, Gonbad Plain, Chemistry, Environmental sciences, Hydrology

## Abstract

Groundwater quality in arid and semi-arid regions is under increasing pressure from both natural and anthropogenic sources. This study presents a comprehensive assessment of groundwater quality in the Gonbad Plain, Iran, to determine its suitability for drinking and to identify the controlling hydrogeochemical processes. A total of 45 groundwater samples were analyzed for their physicochemical parameters, and the data were evaluated using an integrated approach combining hydrochemical facies analysis, geochemical modeling (PHREEQC), multivariate statistical techniques (Principal component analysis (PCA) and hierarchal cluster analysis (HCA)), and human health risk assessment (Heavy metal pollution index (HPI), Hazard quotient (HQ), Hazard index (HI)). The results reveal a hydrochemical evolution from fresh Ca-HCO₃ type water to saline Na-Cl type water, primarily controlled by three key mechanisms: (1) dissolution of evaporite minerals (halite and gypsum), (2) cation exchange, and (3) evaporation. The Gibbs diagrams confirmed that rock-water interaction and evaporation are the dominant processes governing water chemistry. Iron concentrations varied from 0.01 mg/L to 4.12 mg/L, with a mean of 0.46 mg/L and a standard deviation of 0.66 mg/L. The median iron concentration was 0.27 mg/L. Manganese concentrations ranged from 0.015 mg/L to 0.47 mg/L, with a mean of 0.09 mg/L and a standard deviation of 0.07 mg/L. The median manganese concentration was 0.08 mg/L. The HPI indicated a critical water quality crisis, with 77.8% of the samples being unsuitable for drinking (HPI > 100), primarily due to elevated concentrations of manganese. The non-carcinogenic health risk assessment revealed that while the dermal exposure pathway poses no significant risk, oral ingestion of groundwater presents a potential health hazard, particularly for children. The HI exceeded the safe limit of 1.0 in 11.1% of the samples for children, with manganese being the primary contributor to the risk. This is the first study apply integrated framework that combines multivariate statistics, geochemical modeling, and health risk indices to evaluate groundwater vulnerability in Gonbad-e Kavus. The study’s alignment with UN Sustainable Development Goal 6 (Clean Water and Sanitation).

**Supplementary Information:**

The online version contains supplementary material available at 10.1038/s41598-026-51636-2.

## Introduction

Groundwater is a critical global resource, with approximately one-third of the world’s population relying on it as their primary source of drinking water^1^. However, the global groundwater quality crisis is accelerating due to a combination of geogenic processes and anthropogenic pressures, leaving an estimated 1.7 billion people exposed to contaminated drinking water sources^2^. In arid and semi-arid regions, such as the Middle East, this crisis is particularly acute. In Iran, rapid population growth and intensive agricultural expansion have led to severe groundwater depletion and widespread salinization across multiple alluvial aquifers^3,4^. Within the Golestan Province of northern Iran, the Gonbad Plain represents a highly vulnerable semi-arid aquifer system where the interplay between natural hydrogeological conditions and human activities threatens the sustainability of the drinking water supply.

Understanding the hydrochemical evolution of such aquifers requires a detailed assessment of rock-water interactions. As groundwater flows through the aquifer matrix, its chemical composition is continuously modified by processes including the dissolution of evaporites (e.g., halite and gypsum), the weathering of primary silicate minerals, and cation exchange^5–7^. While heavy metal contamination is a recognized global health risk^8–10^, the specific trace element profile of an aquifer is heavily dependent on local lithology and redox conditions. In the Gonbad Plain, preliminary surveys indicated that iron (Fe) and manganese (Mn) are the primary trace metals mobilized from the clay-rich alluvial sediments. Consequently, this study focuses specifically on the non-carcinogenic health risks associated with Fe and Mn exposure. While other toxic metals (e.g., As, Pb, Cd, Cr) are critical pollutants in many Iranian aquifers^11^, they were not included in the current analytical scope and remain a priority for future investigations.

The assessment of groundwater quality is a complex, multi-faceted process that requires an integrated approach. Hydrochemical evaluation, which involves the analysis of the chemical composition of water, is the first step in understanding the processes that control water quality^12–15^. This evaluation often employs a variety of graphical and statistical methods to identify the dominant hydrochemical facies and the sources of dissolved ions. However, a simple chemical analysis is often insufficient to fully understand the complex interactions between water and the aquifer minerals. Geochemical modeling, using tools such as PHREEQC, provides a more in-depth understanding of these interactions^7,16,17^. By calculating mineral saturation indices (SI), geochemical models can predict the tendency of minerals to dissolve or precipitate, thereby revealing the key geochemical processes, such as weathering, ion exchange, and dissolution of salts, that govern the water’s chemistry^18,19^.

In recent years, multivariate statistical techniques, such as Principal Component Analysis (PCA) and Hierarchical Cluster Analysis (HCA), have become indispensable tools in water quality assessment^20,21^. PCA is a powerful technique for reducing the dimensionality of complex datasets, identifying the dominant factors that explain the variance in water quality, and revealing the relationships between different physicochemical parameters^5,22^. HCA, on the other hand, is used to group water samples into distinct clusters based on their chemical similarities, which can help to delineate different hydrochemical zones and identify areas with similar pollution sources^23^. The combined use of PCA and HCA provides a robust framework for interpreting complex water quality data and understanding the spatial patterns of contamination.

The presence of heavy metals in drinking water, even at trace levels, can have severe health implications. Iron (Fe) and manganese (Mn), while being essential elements at low concentrations, can be toxic at higher levels^24,25^. Chronic exposure to high concentrations of manganese in drinking water has been linked to neurotoxic effects, particularly in children, leading to problems with memory, attention, and motor skills^26^. Therefore, a comprehensive assessment of groundwater quality must include a thorough evaluation of the health risks associated with heavy metal contamination.

To quantify the overall pollution status of water with respect to heavy metals, the Heavy Metal Pollution Index (HPI) is a widely used tool^21,22^. The HPI provides a single, aggregate value that reflects the combined effect of different heavy metals, allowing for a straightforward comparison of the pollution levels of different water sources. An HPI value above 100 is generally considered to indicate that the water is unsuitable for drinking^27,28^.

For a more detailed assessment of the potential non-carcinogenic health risks posed by specific contaminants, the Hazard Quotient (HQ) and Hazard Index (HI) are used, following the methodology proposed by the United States Environmental Protection Agency (USEPA)^20,29,30^. The HQ is the ratio of the potential exposure to a single substance to the level at which no adverse health effects are expected. The HI is the sum of the HQs for multiple substances and/or multiple exposure pathways. An HI value greater than 1.0 suggests that there is a potential for adverse non-carcinogenic health effects^31^.

While comprehensive heavy metal assessments are ideal, preliminary regional screenings in the Gonbad Plain identified Iron (Fe) and Manganese (Mn) as the primary trace metals of concern, often mobilized by specific redox conditions and agricultural practices^32^. Elevated manganese in drinking water is of particular public health concern due to its documented neurotoxic effects, especially in children, leading to developmental and cognitive impairments^33^. Despite the critical reliance on the Gonbad Plain aquifer, there remains a significant research gap: a lack of an integrated approach that simultaneously evaluates the hydrogeochemical evolution mechanisms and quantifies the specific health risks posed by these mobilized metals. Addressing this gap is essential for advancing the targets of UN Sustainable Development Goal 6 (Clean Water and Sanitation) in vulnerable semi-arid regions.

Therefore, the specific objectives of this study are to: (1) Determine the spatial distribution and statistical variation of physicochemical parameters in the Gonbad Plain groundwater. (2) Identify the dominant hydrogeochemical facies and the natural mechanisms controlling groundwater evolution using Piper and Gibbs diagrams, supported by PHREEQC geochemical modeling. (3) Delineate the primary sources of contamination (natural vs. anthropogenic) using multivariate statistical techniques (PCA and HCA). (4) Evaluate the suitability of groundwater for drinking purposes by comparing major ions and salinity against WHO standards, and quantify the specific non-carcinogenic health risks posed by Fe and Mn exposure using the Heavy Metal Pollution Index (HPI) and Hazard Index (HI).

## Materials and methods

### Study area description

**Fig. 1:**
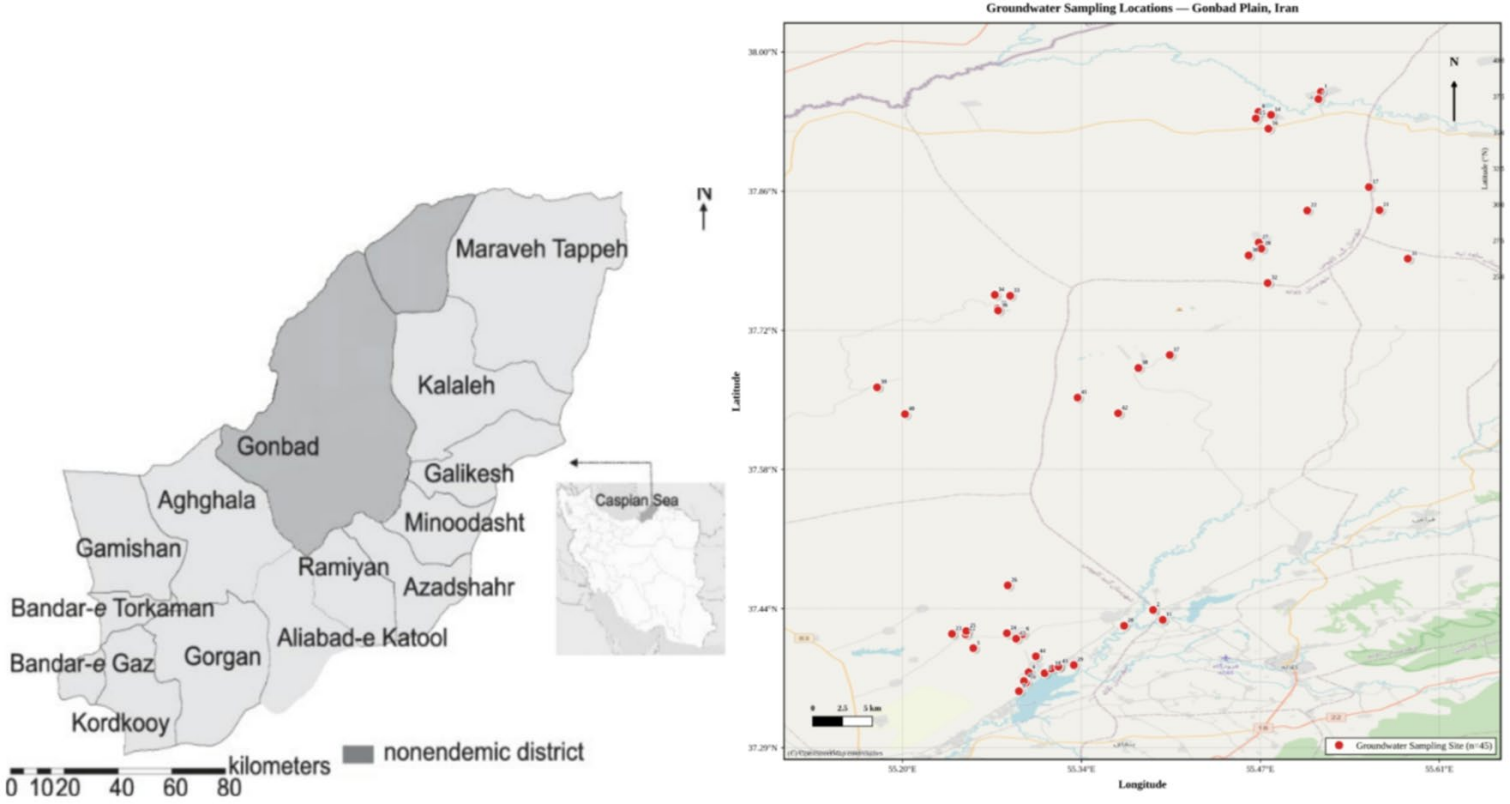
Location map and sampling sites of the investigated area. The map was generated using QGIS (version 3.42.0;https://www.qgis.org/project/visualchangelogs/visualchangelog342/) with basemap data from OpenStreetMap contributors.

The study area is situated in the northeastern part of Golestan Province, Iran, between 37°36′–37°58′ N and 55°18′–55°58′ E. The Gonbad Plain, covering a total surface area of approximately 4,200 square kilometers, is located in the central part of the Golestan Province in northern Iran Fig.1. The plain is bounded by the Alborz Mountain range to the south and extends towards the semi-arid steppes in the north. It occupies the central to northeastern portion of the Gonbad-e Kavous Plain, lying administratively within Gonbad-e Kavous County. The area extends northward from Gonbad-e Kavous city toward the village of Korand, reaching the Iran–Turkmenistan border. It is bounded by the Atrak River basin and Maraveh Tappeh to the east, and by the middle sectors of the Gorgan Plain to the west.

Topographically, the plain represents a gently sloping alluvial surface formed during the Quaternary period, descending from the foothills of the eastern Alborz Mountains in the south toward the Atrak River lowlands in the north. Elevation ranges from approximately 120 m in the southern part to about 60 m above mean sea level near the northern boundary. The area forms part of the Gorganrud–Atrak interfluvial plain, characterized by extensive alluvial and fluvial deposits and intensive agricultural activities^34^. The region experiences a semi-arid, hot steppe climate (Köppen–Geiger BSh) characterized by hot, dry summers and mild winters. Mean annual temperatures range from 17 °C to 18.5 °C, with annual precipitation varying between 350 and 400 mm, concentrated primarily during winter and early spring. A critical climatic feature of the plain is the severe evaporation–precipitation imbalance; mean annual potential evaporation (1,800–2,000 mm) exceeds rainfall by four to five times. This high evaporation rate, occasionally exacerbated by strong northeastern winds, is a primary driver of soil and groundwater salinization, particularly in the northern sectors of the plain^35,36^.

#### Geological and hydrogeological setting

The study area is located on the southern margin of the Kopet-Dagh structural zone in the Gonbad alluvial plain, formed during the Quaternary period by extensive fluvial sedimentation at the northern foothills of the eastern Alborz Mountains^37^. The surface geology is dominated by unconsolidated Quaternary deposits (sand, silt, and clay) that reach thicknesses of 40 to 120 m. These deposits form the primary unconfined to semi-confined aquifer system of the region, underlain by the Neogene Gorgan Formation (marl, sandstone, and limestone) which acts as the hydrogeological basement^38,39^.

The aquifer exhibits significant spatial heterogeneity. The southern portion near Gonbad-e Kavous is characterized by coarser-grained alluvial sediments with higher hydraulic conductivity and transmissivity. Moving northward, the sediments gradually transition into finer-grained deposits with increasing clay content, accompanied by the local presence of gypsum and halite lenses reflecting evaporitic conditions^40,41^.

Groundwater flow generally follows the regional topographic gradient from the southern and southeastern elevated recharge zones toward the northern and northwestern lowlands, eventually discharging into the Atrak River basin^42,43^. The water table is relatively shallow, ranging from 5 to 20 m below the ground surface. Recharge primarily occurs through rainfall infiltration over the southern piedmont areas, subsurface inflow from the Alborz foothills, and percolation from agricultural irrigation return flows^44^. The combination of a shallow water table, fine-grained northern sediments, and high evaporation rates facilitates the accumulation of salts in the northern plain, significantly influencing the hydrochemical evolution of the groundwater^45^.

Due to limited precipitation and the absence of perennial surface water, this aquifer serves as the primary and often sole reliable water source for the Gonbad Plain^46^. Approximately 70–80% of extracted groundwater is utilized for agriculture, where traditional flood irrigation practices contribute to significant evaporative losses and subsequent waterlogging and salinization in low-lying northern areas^47^.

### Sampling and physicochemical analysis

A total of 45 groundwater samples were collected from representative wells across the Gonbad Plain during the dry season of 2023. The samples were collected from deep bore wells (tube wells) utilized for both agricultural irrigation and domestic water supply. The depths of the sampled wells range from 40 to 120 m, tapping into the primary confined and semi-confined alluvial aquifer units. Prior to sampling, each well was purged for a minimum of 15 min until field parameters stabilized, to ensure that the collected water was representative of the aquifer formation. In-situ measurements of temperature (T), pH, and electrical conductivity (EC) were performed at each sampling site using a calibrated portable multi-parameter probe (Hanna Instruments HI9829). The samples were collected in 1-liter pre-cleaned, high-density polyethylene (HDPE) bottles, which were rinsed three times with the sample water before being filled.

Following collection, the samples were immediately placed in ice-cooled boxes and transported to the laboratory for detailed chemical analysis within 24 h. The concentrations of major cations (Ca²⁺, Mg²⁺, Na⁺, K⁺) and trace metals (Fe, Mn) were determined using Atomic Absorption Spectrophotometry (AAS). The major anions were analyzed using various standard techniques: chloride (Cl⁻) was determined by the argentometric titration method, bicarbonate (HCO₃⁻) was measured by acid titration, sulfate (SO₄²⁻) was analyzed using the turbidimetric method, and nitrate (NO₃⁻) was determined spectrophotometrically. Total Dissolved Solids (TDS) were measured by the gravimetric method after evaporation at 180 °C. All chemical concentrations were reported in milligrams per liter (mg/L), with the exception of EC, which was reported in microsiemens per centimeter (µS/cm), pH (unitless), and temperature (°C).

To ensure data reliability, stringent quality assurance and quality control (QA/QC) procedures were implemented. Reagents were prepared using deionized water, and instruments were calibrated with certified standard solutions prior to analysis. One duplicate and one blank sample were analyzed for every ten samples to monitor precision and potential contamination. Analytical precision was verified by calculating the ionic balance error (IBE) for each sample (Eq. 1).

1 $$\:IBE\left(\%\right)=\left(\frac{\sum\:Cations-\sum\:Anions}{\sum\:Cations+\sum\:Anions}\right)*100$$

where all ionic concentrations are expressed in milliequivalents per liter (meq/L). All samples analyzed in this study showed an ionic balance error within the acceptable limit of ± 5%, confirming the high quality and reliability of the analytical results.

### Geochemical modeling

To determine the thermodynamic equilibrium state of the groundwater with respect to various mineral phases, geochemical modeling was performed using the PHREEQC software^5,19,48^. The software calculates the Saturation Index (SI) for each mineral, which indicates the tendency of the water to dissolve or precipitate that mineral. The SI is calculated using the following equation (Eq. 2):

2 $$\:SI=Log\:\left(\frac{IAP}{Ksp}\right)$$

where IAP is the Ion Activity Product of the mineral’s constituent ions in the solution, and Ksp is the solubility product of the mineral at the measured temperature. The interpretation of the SI values is as follows:

- SI > 0: The water is supersaturated with respect to the mineral, and precipitation is likely to occur.
- SI < 0: The water is undersaturated with respect to the mineral, and it has the capacity to dissolve more of that mineral.
- SI = 0: The water is in equilibrium with the mineral.

This analysis was crucial for identifying the key geochemical processes, such as the dissolution of evaporites (gypsum, halite) and the precipitation of carbonates (calcite, dolomite), that control the groundwater chemistry in the study area.

### Multivariate statistical analysis

Multivariate statistical analyses, including Principal Component Analysis (PCA) and Hierarchical Cluster Analysis (HCA), were performed using the Python programming language with the scikit-learn and SciPy libraries to identify the underlying factors controlling groundwater quality and to group samples with similar Hydrochemical characteristics. Prior to analysis, the dataset, comprising 45 samples and 15 physicochemical parameters, was standardized (z-score transformation) to normalize the data and prevent misclassification due to differences in measurement units^49^.

PCA was applied to reduce the dimensionality of the dataset and to extract the principal components (PCs) that explain the majority of the variance in the data. The suitability of the data for PCA was confirmed using the Kaiser-Meyer-Olkin (KMO) and Bartlett’s sphericity tests. PCs with eigenvalues greater than 1 (Kaiser’s criterion) were considered significant and were retained for further analysis. A Varimax rotation was applied to the PCs to maximize the variance of the factor loadings and facilitate interpretation^50^.

HCA was then performed on the extracted principal component scores to classify the groundwater samples into distinct hydrochemical groups. Ward’s linkage method with Euclidean distance was used as the clustering algorithm, which minimizes the total within-cluster variance. The results of the HCA were visualized as a dendrogram, with the optimal number of clusters determined by observing the largest distance between merged clusters^51^.

### Human health risk assessment

A human health risk assessment was conducted to evaluate the potential non-carcinogenic risks to the local population from exposure to heavy metals (Fe and Mn) in the groundwater, following the guidelines of the United States Environmental Protection Agency (USEPA)^5,24,29,30^. The assessment considered two primary exposure pathways: oral ingestion and dermal contact, for two different population groups: adults and children. The risk assessment involves a two-step process: calculating the Chronic Daily Intake (CDI) and then determining the Hazard Quotient (HQ) and Hazard Index (HI).

The CDI for oral ingestion was calculated using Eq. 3:

3 $$\:CDI\:\left(oral\right)=\frac{C*IR*EF*ED}{BW*AT}$$

The CDI for dermal contact was calculated using Eq. 4:

4 $$\:CDI\:\left(dermal\right)=\frac{C*SA*Kp*ET*CF*EF*ED}{BW*AT}$$

where all parameters and their corresponding values are defined in Table 1.

**Table 1:** Parameters used for the health risk assessment.

Parameter	Description	Unit	Adult	Child	Reference
C	Concentration of metal in water	mg/L	Measured	Measured	This study
IR	Ingestion Rate	L/day	2.2	1.5	^5^
EF	Exposure Frequency	days/year	365	365	^9^
ED	Exposure Duration	years	30	6	^24^
BW	Body Weight	kg	70	15	^13^
AT	Averaging Time (non-carcinogenic)	days	10,950	2190	^52^
SA	Skin Surface Area	cm²	18,000	6600	^10^
Kp	Dermal Permeability Coefficient	cm/h	0.001 (Fe), 0.0004 (Mn)	0.001 (Fe), 0.0004 (Mn)	^10^
ET	Exposure Time	h/day	0.6	1	^10^
CF	Conversion Factor	L/cm³	0.001	0.001	^52^

The HQ for each metal and exposure pathway was then calculated by dividing the CDI by the corresponding oral or dermal reference dose (RFD), as shown in Eq. 5:

5 $$\:HQ=\frac{CDI}{RFD}$$

The RfD values used were 0.7 mg/kg/day for Fe and 0.14 mg/kg/day for Mn^10^.

Finally, the total potential non-carcinogenic risk is represented by the HI, which is the sum of the HQs for all contaminants and exposure pathways (Eq. 6):

6 $$\:HI=\sum\:HQ$$

An HI value greater than 1.0 indicates a potential for adverse non-carcinogenic health effects, while an HI value of 1.0 or less suggests that adverse health effects are unlikely^52^.

### Heavy metal pollution index (HPI)

To evaluate the overall pollution of groundwater with respect to heavy metals, the HPI was calculated. The HPI is a rating system that provides a composite score based on the weighted average of individual heavy metal concentrations relative to their standard permissible limits^53^. The HPI was calculated using the following two-step process:

First, the sub-index (Qi) for each metal was calculated using Eq. 7:

7 $$\:{Q}_{i}={\sum\:}_{i=1}^{n}100*\left(\frac{{c}_{i}}{Si}\right)$$

Second, the HPI was calculated as the weighted sum of the sub-indices, as shown in Eq. 8:

8 $$\:HPI=\:{\sum\:}_{i=1}^{n}\mathrm{W}\mathrm{i}\mathrm{Q}\mathrm{i}\:/\:{\sum\:}_{i=1}^{n}\mathrm{W}\mathrm{i}$$

where *ci* is the monitored concentration of the ith metal (mg/L), and Si is the standard permissible limit for the ith metal in drinking water, based on WHO guidelines^2^. Wi is the unit weightage for the ith metal, which is defined as the inverse of its standard permissible limit (Wi = 1/Si). The water quality classification based on the calculated HPI value is presented in Table 2.

**Table 2:** HPI Classification for drinking water quality.

HPI Value	Classification
< 25	Excellent
26–50	Good
51–75	Poor
76–100	Very Poor
> 100	Unsuitable for Drinking

### Ethics statement

This study did not involve human participants, human data, or animal experiments. Therefore, ethical approval and informed consent were not required.

### Sampling permission statement

All water samples were collected from publicly accessible groundwater wells located in non-protected areas. No restricted or privately controlled sites were involved in this study, and no specific permission was required for sample collection.

## Results and discussion

### Statistical description and spatial variability of groundwater quality

**Fig. 2:**
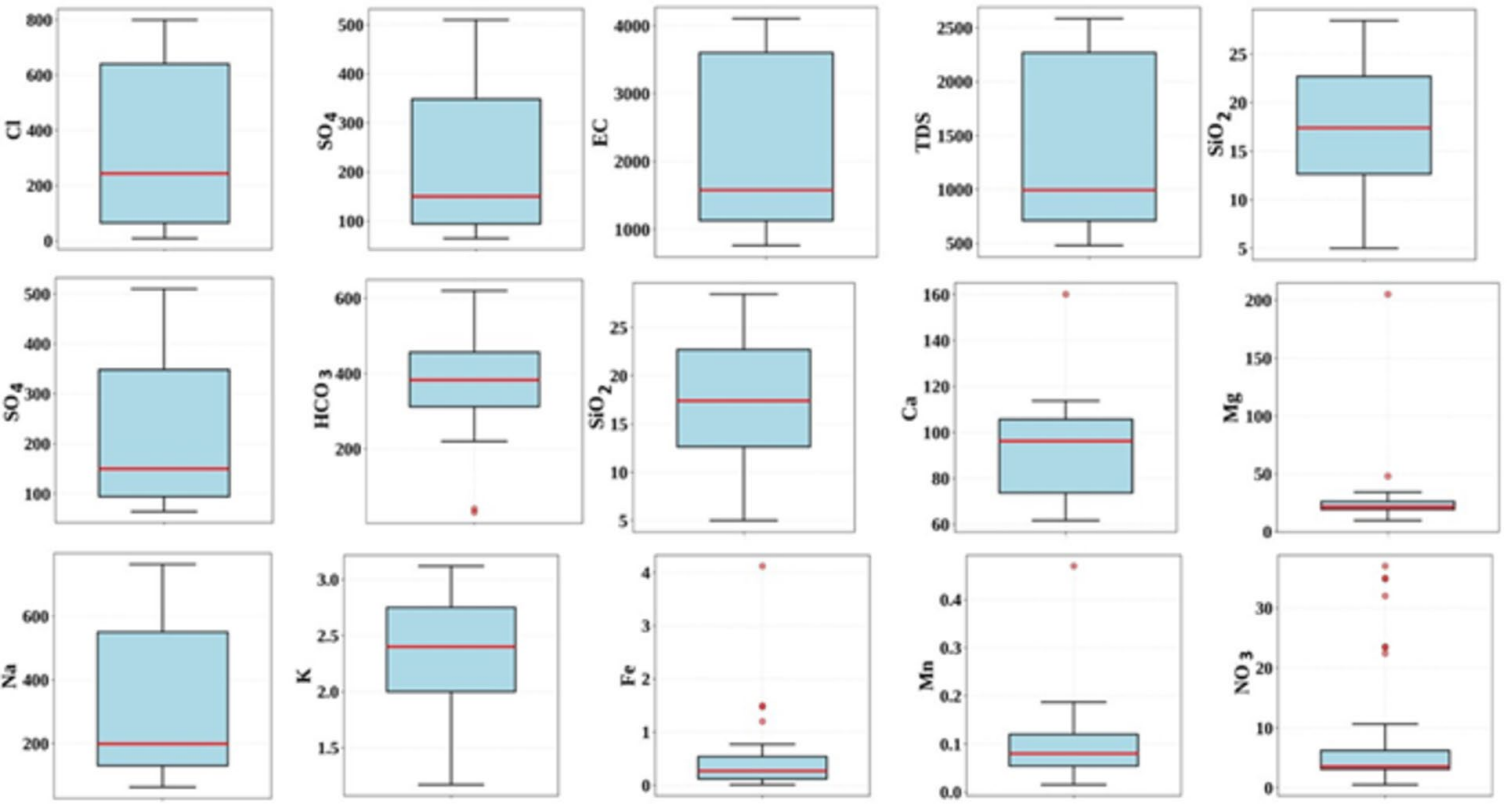
Box plots of major physicochemical parameters in the groundwater of the Gonbad Plain. The plots illustrate the statistical distribution (median, quartiles, and outliers) for T, pH, EC, TDS, and major ions (Cl⁻, SO₄²⁻, HCO₃⁻, Ca²⁺, Mg²⁺, Na⁺, K⁺), as well as trace elements (Fe, Mn) and other parameters (SiO₂, NO₃⁻) for the 45 groundwater samples.

**Fig. 3:**
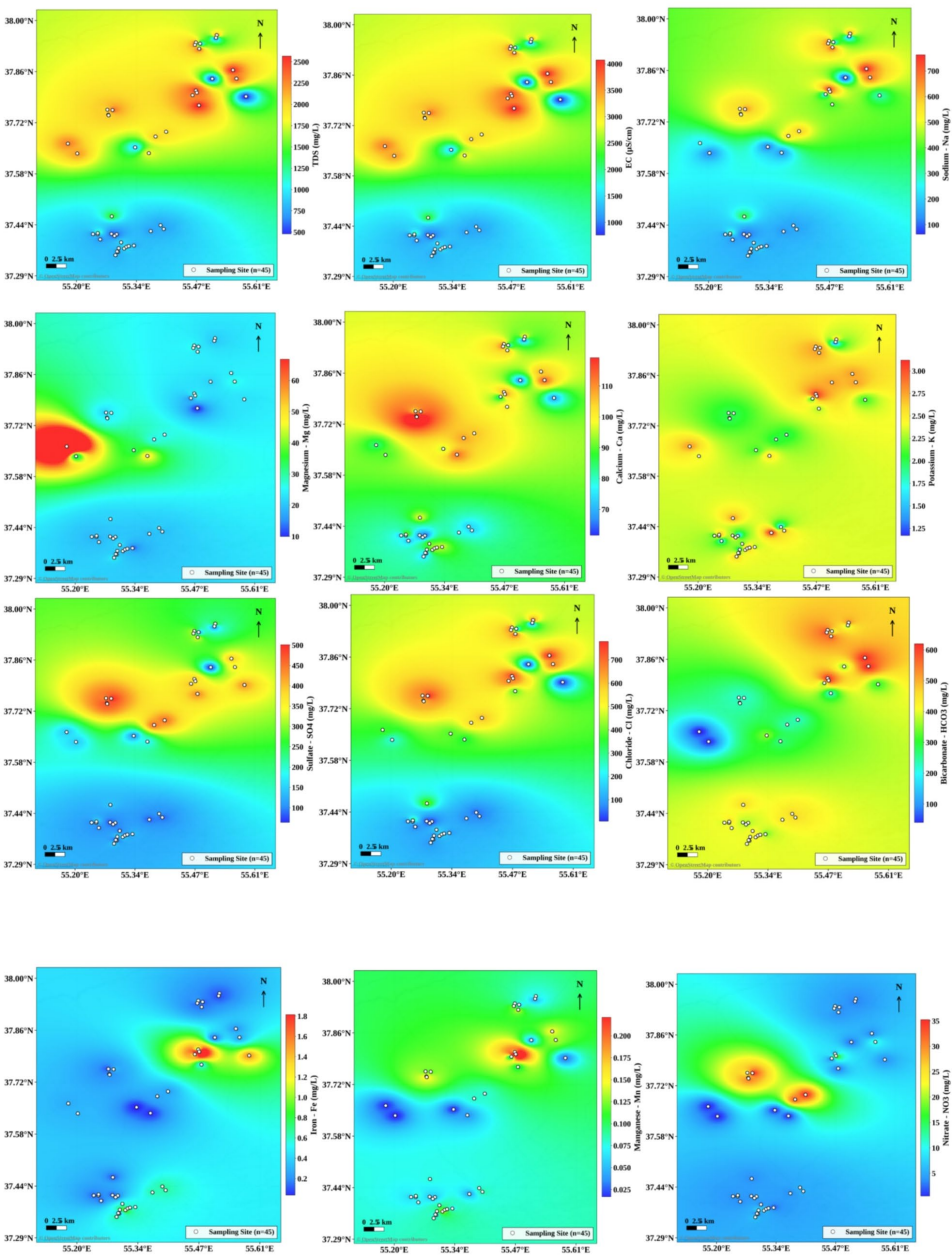
Distribution map of the measured parameters using inverse distance method (IDW).

This section presents a detailed statistical analysis of the physicochemical parameters (Table 1S, Fig. 2) of the groundwater samples collected from the Gonbad region and distribution maps of the measured parameters (Fig. 3). The analysis encompasses descriptive statistics, including the mean, standard deviation, minimum, maximum, and quartiles for each measured parameter. To visually represent the data distribution and identify potential outliers, box plots have been generated for each parameter.

The temperature of the groundwater samples exhibited a considerable range, from a minimum of 8.0 °C to a maximum of 26.8 °C. The mean temperature was calculated to be 21.42 °C, with a standard deviation of 4.79 °C, indicating a moderate level of temperature variation across the sampling sites. The median temperature was 24.0 °C, and the interquartile range (IQR), which represents the middle 50% of the data, spanned from 17.5 °C to 24.9 °C.

The physicochemical parameters of the 45 groundwater samples collected from the Gonbad Plain are statistically summarized in Table 1S, and their spatial distributions are illustrated via Inverse Distance Weighting (IDW) interpolation maps in Fig. 3. Rather than exhibiting a uniform hydrochemical signature, the aquifer demonstrates pronounced spatial heterogeneity driven by a combination of natural lithological interactions, evaporation, and localized anthropogenic inputs.

The pH of the groundwater samples ranged from 7.10 to 7.70 (mean: 7.48), indicating a predominantly slightly alkaline environment. This parameter exhibited minimal spatial variation (Coefficient of Variation, CV = 2.3%), and all samples remained within the World Health Organization (WHO) permissible limits (6.5–8.5) for drinking water. In contrast, salinity indicators exhibited extreme variability and a distinct spatial gradient. The EC ranged from 760 to 4100 µS/cm (mean: 2214.5 µS/cm), and TDS varied from 478.8 to 2583.0 mg/L (mean: 1395.1 mg/L). Both parameters showed high spatial heterogeneity (CV = 55.4%), with 53.3% of samples exceeding the WHO^2^ limit for EC (1500 µS/cm) and 48.9% exceeding the TDS limit (1000 mg/L). As depicted in the spatial distribution maps (Fig. 3), a pronounced south-to-north salinity gradient exists. Samples in the northern sector exhibit mean EC and TDS values more than twice as high as those in the southern recharge zones (e.g., northern mean TDS = 1883.1 mg/L vs. southern mean TDS = 884.9 mg/L). This significant enrichment in salinity toward the north is primarily driven by intense evaporation in the low-lying agricultural areas, where the water table is shallower, coupled with the progressive dissolution of evaporitic minerals along the groundwater flow path.

The spatial distribution of major ions further elucidates these hydrochemical processes. Chloride (Cl⁻) and sodium (Na⁺) were the dominant ions, with mean concentrations of 323.6 mg/L and 319.9 mg/L, respectively. Consequently, 46.7% of the samples exceeded the WHO limits for both Cl⁻ (250 mg/L) and Na⁺ (200 mg/L). Spatially, chloride concentrations in the northern sector were approximately four times higher than in the south (513.3 mg/L vs. 125.4 mg/L). Sulfate (SO₄²⁻) also showed significant spatial enrichment (mean: 222.3 mg/L), with 37.8% of samples exceeding the WHO limit (250 mg/L). The spatial map for SO₄²⁻ mirrors that of TDS, indicating that the dissolution of gypsum within the aquifer matrix is a major contributor to the hydrochemical evolution in the northern plain. Conversely, bicarbonate (HCO₃⁻) was abundant (mean: 385.0 mg/L). Its spatial distribution is more uniform and independent of the salinity gradient, suggesting its origin is primarily linked to abundant carbonate weathering rather than the localized evaporative processes controlling salinity. Magnesium (Mg²⁺) concentrations exhibit the highest spatial variability among the major cations, ranging from 9.60 to 205.00 mg/L, with a mean of 26.62 mg/L and a highly skewed distribution (CV = 106.0%). The vast majority of the aquifer (97.7% of samples) maintains Mg²⁺ concentrations well below the WHO permissible limit of 50 mg/L, typically fluctuating within a narrow interquartile range of 19.2 to 26.1 mg/L. This stable baseline is controlled by the congruent dissolution of carbonate minerals (calcite and dolomite) in the southern recharge zone. However, the extreme maximum value (205.00 mg/L) is driven by a single, highly localized outlier (Sample 39) located in the northern discharge zone. In this specific well, the Mg²⁺/Ca²⁺ molar ratio spikes to 4.01, accompanied by elevated salinity (EC = 3600 µS/cm) and chloride (368 mg/L), but anomalously low bicarbonate (32 mg/L). This distinct hydrochemical signature indicates that the extreme Mg²⁺ enrichment is not a widespread aquifer characteristic, but rather the result of localized dissolution of Mg-bearing evaporite lenses interbedded within the northern playa sediments, combined with intense evaporative concentration in the shallow water table.

Among the trace elements, iron (Fe) and manganese (Mn) exhibited the highest variability in the dataset (CV = 142.7% and 77.4%, respectively). Iron concentrations ranged from 0.01 to 4.12 mg/L (mean: 0.46 mg/L), with 42.2% of the samples exceeding the WHO limit of 0.3 mg/L. The spatial interpolation map for Fe reveals localized hotspots of high concentration in the central and southern areas, which do not align with the northern salinity gradient. These elevated and highly variable concentrations suggest localized reducing conditions within the aquifer, potentially exacerbated by the degradation of agricultural organic matter, which facilitates the mobilization of Fe and Mn from the soil matrix into the groundwater. In contrast, nitrate (NO₃⁻) concentrations (mean: 7.96 mg/L) remained well below the WHO limit of 50 mg/L in all samples. Although NO₃⁻ values are slightly higher in the north (mean: 11.43 mg/L) compared to the south (mean: 4.34 mg/L) due to agricultural return flows, the absolute concentrations indicate that direct nitrate contamination is currently limited, and the primary anthropogenic impact of agriculture in this region manifests as secondary salinization rather than nutrient pollution.

### Multivariate statistical analysis

To better understand the complex relationships between the physicochemical parameters and to identify the main factors controlling the groundwater chemistry in the Gonbad Plain, PCA and HCA were performed on the dataset of 45 groundwater samples and15 parameters.

**Fig. 4:**
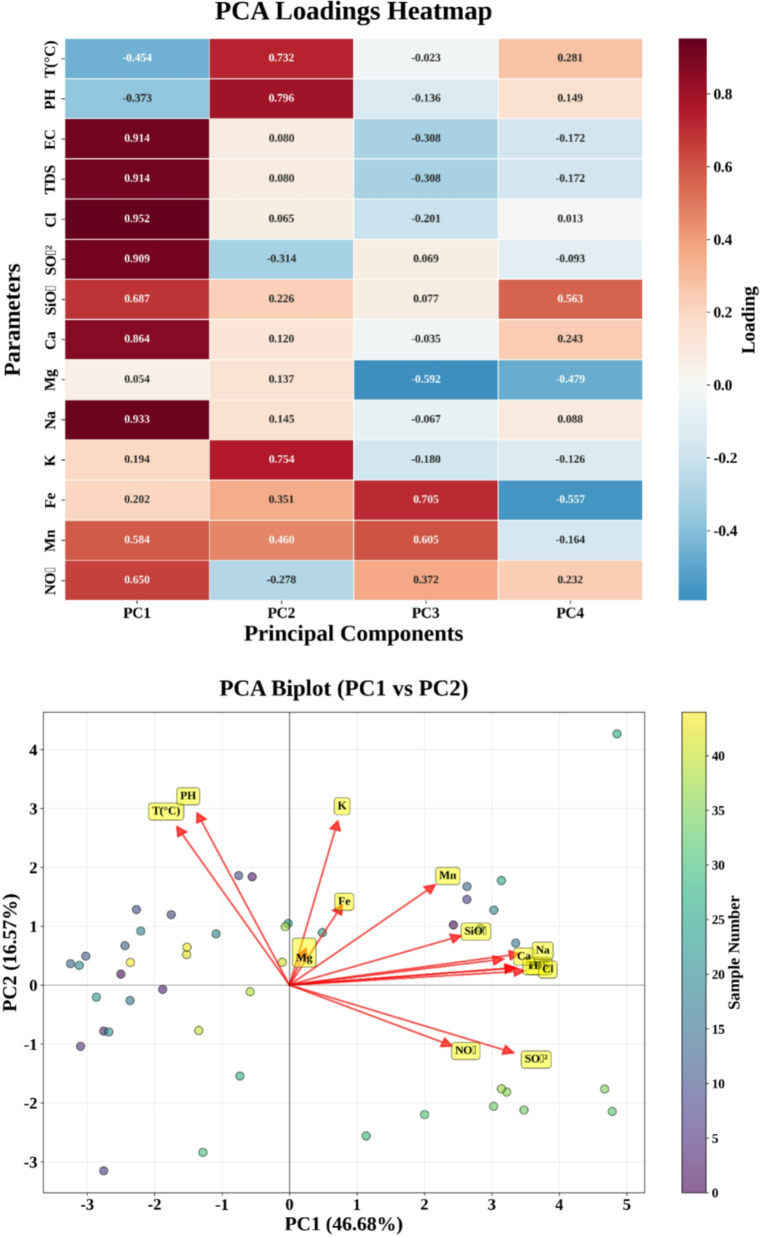
Principal Component Analysis (PCA) heat map and biplot of the hydrochemical data. The plot shows the scores of the 45 groundwater samples and the loadings of the 15 physicochemical parameters on the first two principal components (PC1 and PC2). PC1 represents the salinity component, while PC2 reflects a combination of agricultural and geochemical influences.

Principal Component Analysis (Fig. 4) with heat map and biplot was applied to the standardized dataset to reduce the dimensionality of the data and to identify the dominant hydrogeochemical processes. Based on the Kaiser criterion (eigenvalues > 1), four principal components (PCs) were extracted, which collectively explain 72.84% of the total variance in the dataset. The first two components, PC1 and PC2, account for 46.68% and 16.57% of the variance, respectively, making them the most significant for interpretation.

**PC1: The Salinity Component**

PC1, explaining nearly half of the total variance, is strongly and positively correlated with parameters indicative of salinity: EC, TDS, Cl⁻, Na⁺, and SO₄²⁻. The high positive loadings for these variables (Fig. 4) indicate that PC1 represents the overall mineralization and salinity of the groundwater. Samples with high positive scores on PC1 are characterized by high concentrations of dissolved solids, which is a direct reflection of natural salinization processes, such as the dissolution of evaporitic minerals (halite and gypsum) known to be present in the northern part of the study area. This component effectively separates the samples along a salinity gradient, from fresher groundwater (negative scores) to highly saline water (positive scores).

**PC2: The Agricultural and Geochemical Component**

PC2 is characterized by moderate positive loadings for Mn, Fe, and K⁺, and moderate negative loadings for NO₃⁻ and SO₄²⁻. This component appears to represent a mix of influences. The association of nitrate (NO₃⁻) points towards an anthropogenic source, likely related to the extensive use of nitrogen-based fertilizers in the agricultural areas of the Gonbad Plain. The negative loading suggests an inverse relationship with the parameters having positive loadings. The presence of Fe and Mn may be related to redox conditions in the aquifer. The grouping of these variables in PC2 suggests that this component reflects the combined influence of agricultural contamination and specific geochemical conditions within the aquifer.

The PCA biplot of PC1 versus PC2 visually confirms these interpretations. The samples are distributed primarily along the PC1 axis, clearly separating the low-salinity samples on the left from the high-salinity samples on the right. The loading vectors for EC, TDS, Cl⁻, and Na⁺ point strongly to the right, aligned with the high-salinity samples. The NO₃⁻ vector points downwards in the negative PC2 direction, associating it with a different process than the primary salinity.

**Hierarchical Cluster Analysis (HCA)**

**Fig. 5:**
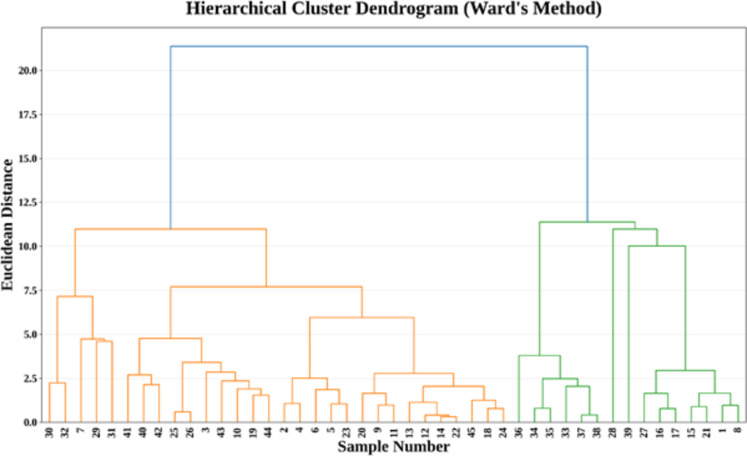
Dendrogram of the HCA of groundwater samples. The dendrogram was generated using Ward’s linkage method with Euclidean distance, illustrating the classification of the 45 groundwater samples into three distinct hydrochemical clusters based on their physicochemical similarities.

**Fig. 6:**
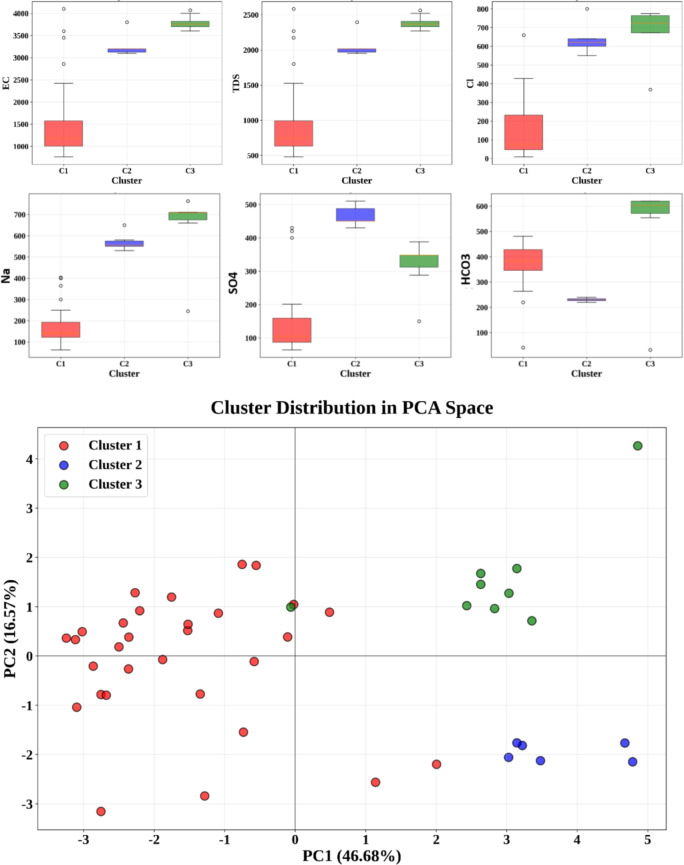
Relationship between clusters and measured parameters and PCA.

Hierarchical Cluster Analysis using Ward’s linkage method and Euclidean distance was performed on the standardized data to group the groundwater samples into distinct hydrochemical clusters. The resulting dendrogram (Fig. 5) suggested the presence of three main clusters, which were further analyzed to understand their spatial distribution and chemical characteristics (Fig. 6).

**Cluster 1: Moderately Mineralized Water**

This is the largest cluster, comprising 30 out of the 45 samples. These samples are characterized by relatively low to moderate salinity, with an average EC of 1534 µS/cm. The concentrations of major ions like Cl⁻ (153 mg/L) and Na⁺ (169 mg/L) are significantly lower than in the other two clusters. Geographically, these samples are likely located in the southern and central parts of the plain, where recharge from the Alborz foothills provides fresher water, and the influence of evaporitic deposits is minimal. This cluster represents the baseline groundwater quality in the less-impacted areas of the aquifer.

**Cluster 2: High-Salinity**,** High-Nitrate Water (Agricultural Impact)**

This cluster consists of 6 samples (33, 34, 35, 36, 37, 38) and is defined by high salinity (average EC of 3267 µS/cm) and exceptionally high concentrations of Nitrate (NO₃⁻), with a mean of 30.77 mg/L. This chemical signature strongly points to an anthropogenic origin, where irrigation return flows carrying excess fertilizers have significantly impacted the groundwater quality. The high levels of other salts (Cl⁻, SO₄²⁻) are likely exacerbated by the dissolution of soil salts due to agricultural practices. These samples are likely located in the northern agricultural zones where drainage is poor.

**Cluster 3: Very High-Salinity Water (Natural Salinization)**

This cluster includes 9 samples and represents the most saline groundwater in the study area, with an average EC of 3781 µS/cm. It is characterized by very high concentrations of Na⁺, Cl⁻, and SO₄²⁻, but with moderate nitrate levels (6.61 mg/L) compared to Cluster 2. This composition is consistent with natural salinization processes, primarily the dissolution of halite (NaCl) and gypsum (CaSO₄·2 H₂O) from the geological formations in the northern part of the plain, where high evaporation rates concentrate the dissolved salts. These samples represent water that has undergone significant water-rock interaction and evaporation.

The combined results of PCA and HCA (Fig. 6) provide a clear picture of the hydrogeochemical processes in the Gonbad Plain. The dominant factor controlling groundwater chemistry is salinity, which increases from south to north. The multivariate analysis successfully distinguished between two primary sources of this high salinity: Natural Salinization driven by the dissolution of evaporitic minerals in the northern part of the plain, leading to very high concentrations of Na⁺, Cl⁻, and SO₄²⁻ (Cluster 3). Anthropogenic Contamination resulting from agricultural activities, which introduces high levels of NO₃⁻ and other salts into the groundwater system (Cluster 2).

The majority of the groundwater (Cluster 1) remains moderately fresh, but the presence of the other two clusters highlights the significant pressures on the aquifer from both natural and human-induced sources. These findings are critical for developing sustainable water management strategies for the Gonbad Plain, which should focus on controlling agricultural pollution and managing the extraction of highly saline water.

### Hydrochemistry and geochemical evaluation

#### PHREEQC model and Saturation index estimation

**Fig. 7:**
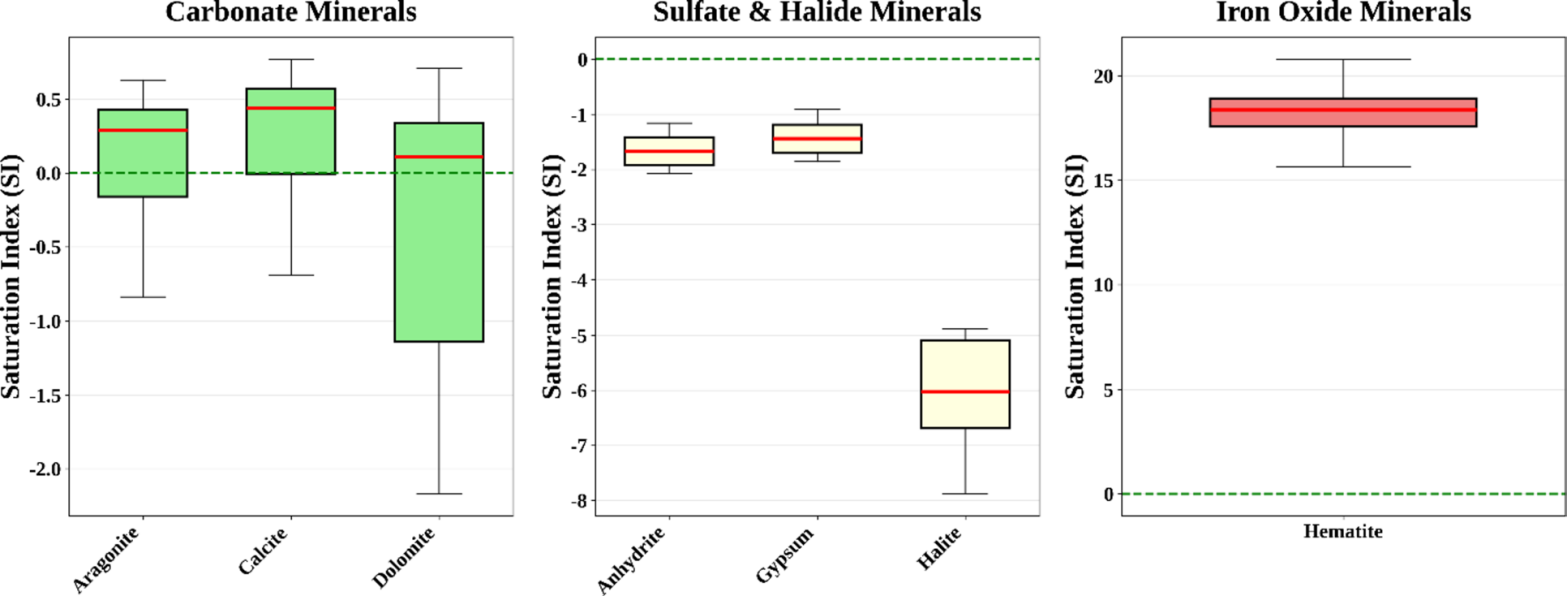
Box plots of the Saturation Index (SI) for major mineral phases in the groundwater. The plots show the SI values for key carbonate (Calcite, Aragonite, Dolomite), sulfate (Anhydrite, Gypsum), halide (Halite), and iron oxide (Hematite) minerals.

To assess the thermodynamic equilibrium state between the groundwater and various mineral phases, the Saturation Index (SI) was calculated for key carbonate, sulfate, halide, and iron oxide minerals using the PHREEQC geochemical modeling software (Fig. 7). The Saturation Index indicates the tendency of a mineral to either precipitate from or dissolve into the water. A positive SI value (SI > 0) indicates supersaturation and a potential for mineral precipitation, a negative value (SI < 0) indicates undersaturation and a potential for mineral dissolution, and an SI value of zero (SI = 0) signifies equilibrium.

**Carbonate Minerals (Calcite**,** Aragonite**,** Dolomite)**

The analysis reveals that the majority of groundwater samples are supersaturated with respect to carbonate minerals. Specifically, 73% of the samples are supersaturated with Calcite (CaCO₃) and 71% with Aragonite (CaCO₃), its polymorph. This indicates a strong thermodynamic potential for the precipitation of calcium carbonate throughout the aquifer system, which is a common characteristic of groundwater in alluvial plains containing carbonate sediments. The mean SI values for Calcite and Aragonite were 0.30 and 0.16, respectively.

Dolomite (CaMg(CO₃)₂) also showed a tendency towards supersaturation in 60% of the samples. However, a significant portion (40%) remained undersaturated, and the overall mean SI for Dolomite was slightly negative (−0.26). This suggests that while the conditions are often favorable for dolomite precipitation, its formation is likely limited by slower precipitation kinetics compared to calcite and aragonite, or by the specific Mg/Ca ratios in the water. The general supersaturation of carbonate minerals suggests that carbonate precipitation is an active process controlling the concentrations of Ca²⁺ and the alkalinity of the groundwater.

**Sulfate and Halide Minerals (Anhydrite**,** Gypsum**,** Halite)**

In contrast to the carbonate minerals, all 45 groundwater samples were found to be 100% undersaturated with respect to the evaporite minerals Anhydrite (CaSO₄), Gypsum (CaSO₄·2 H₂O), and Halite (NaCl). The Saturation Indices for these minerals were strongly negative across the entire dataset, with mean values of −1.67 for Anhydrite, −1.44 for Gypsum, and − 6.02 for Halite.

This finding is highly significant. While the previous multivariate analysis (PCA/HCA) identified the dissolution of these evaporitic minerals as a primary source of salinity in the study area (particularly in Cluster 3), the SI results demonstrate that even the most saline waters have not reached equilibrium with these phases. This implies that the groundwater still has a substantial capacity to dissolve more of these salts. The presence of these minerals in the geological formations acts as a continuous source of salinity as groundwater flows through the plain, but their dissolution is not sufficient to bring the bulk of the water to a state of saturation.

**Iron Oxide Minerals (Hematite)**

Geochemical modeling revealed that the majority of groundwater samples are supersaturated with respect to iron oxide minerals, particularly hematite. While thermodynamic supersaturation of hematite is often associated with oxidizing environments, the elevated concentrations of dissolved Fe (up to 4.12 mg/L) observed in the Gonbad Plain indicate that active iron mobilization is occurring. In alluvial aquifers, such mobilization is typically driven by localized reducing conditions, where the microbial degradation of sedimentary organic matter within clay-rich lenses consumes available oxygen, lowering the redox potential and reducing insoluble Fe(III) oxides to highly soluble Fe(II)^54,55^. Therefore, rather than confirming a uniformly oxidizing aquifer, the PHREEQC saturation indices, combined with the high dissolved Fe concentrations, point to a complex system where geogenic iron is actively mobilized in reducing micro-environments before precipitating as secondary oxides along the flow path.

The Saturation Index analysis highlights the key geochemical controls on the groundwater chemistry of the Gonbad Plain: Carbonate Precipitation: The system is largely at or above saturation with respect to calcite and aragonite, suggesting that carbonate precipitation is a dominant process buffering the water chemistry. Evaporite Dissolution: The system is significantly undersaturated with respect to gypsum and halite, confirming that the dissolution of these minerals is an ongoing and primary source of salinity. Oxidizing Conditions: While hematite supersaturation suggests an oxidizing environment conducive to iron precipitation, it cannot definitively confirm the overall redox state without accompanying Eh/pe measurements.

#### Ionic ratio and mechanism govern water chemistry

**Fig. 8:**
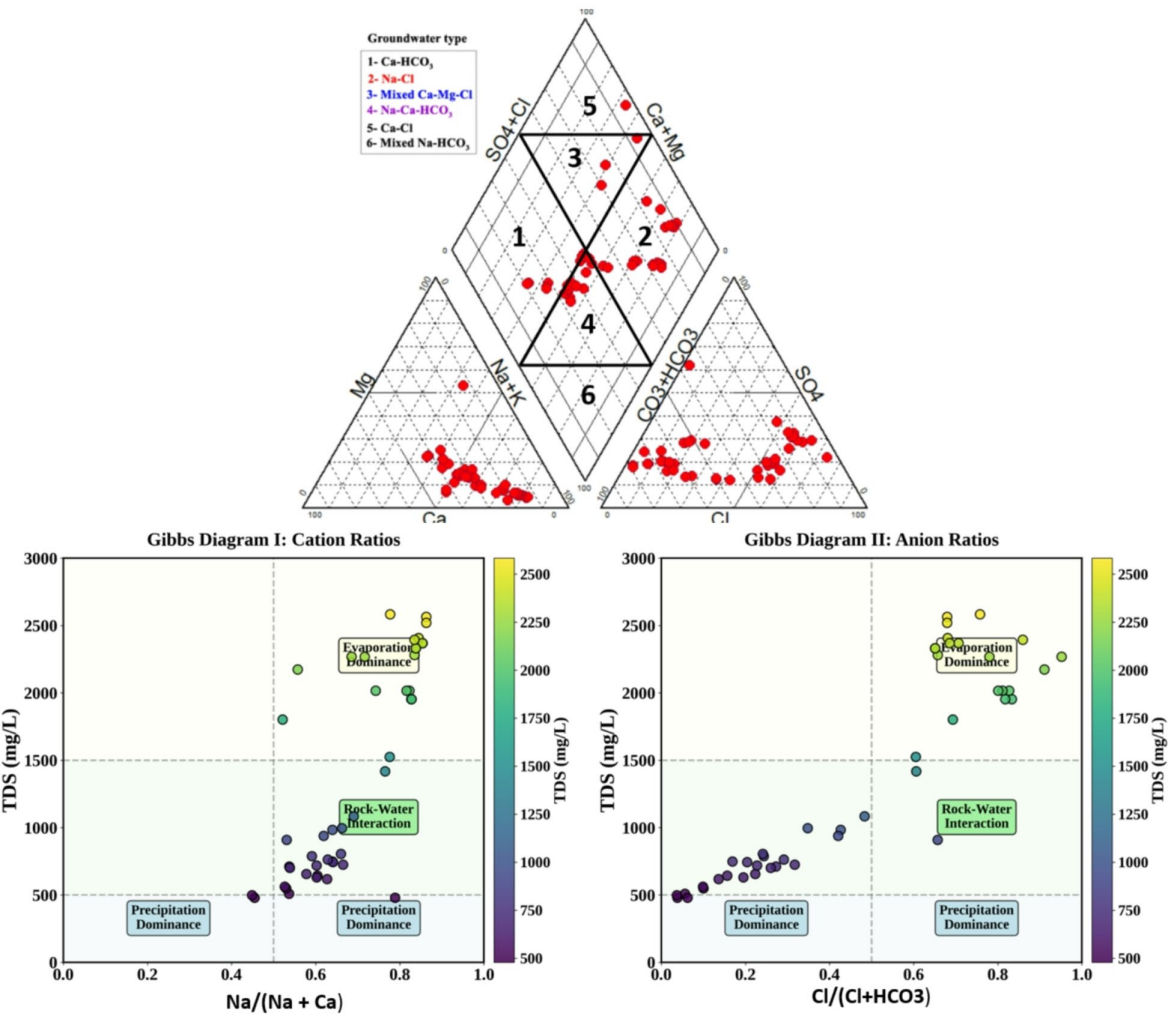
Gibbs diagrams and piper diagram illustrating the mechanisms controlling groundwater chemistry.

**Fig. 9:**
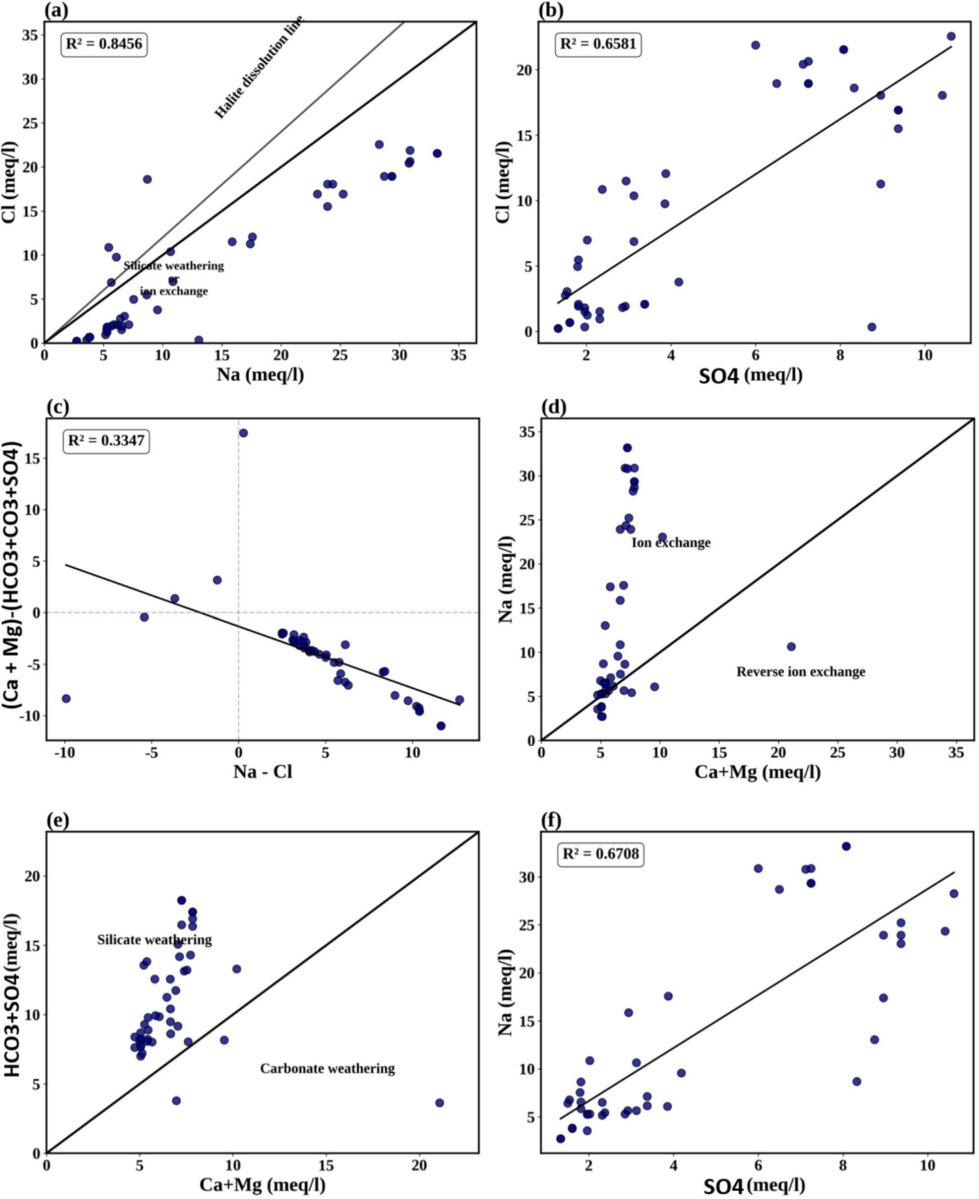
Bivariate plots of major ionic ratios for identifying hydrogeochemical processes. The six-panel figure shows the relationships between (**a**) Cl⁻ vs. Na⁺, (**b**) Cl⁻ vs. SO₄²⁻, (**c**) (Ca²⁺+Mg²⁺)-(HCO₃⁻+SO₄²⁻) vs. (Na⁺-Cl⁻), (**d**) Na⁺ vs. (Ca²⁺+Mg²⁺), (**e**) (SO₄²⁻+HCO₃⁻) vs. (Ca²⁺+Mg²⁺), and (**f**) Na⁺ vs. SO₄²⁻. The plots include 1:1 lines and labels indicating the dominant processes such as halite dissolution, ion exchange, and weathering.

The hydrogeochemical characteristics of the groundwater in the Gonbad Plain were investigated using ionic ratio plots and Gibbs and piper diagrams to identify the primary mechanisms controlling water chemistry. The results, presented in Fig. 8**and** Fig. 9, reveal a complex interplay between rock-water interactions, evaporation, and ion exchange processes, which collectively govern the evolution of groundwater from fresh to saline.

The hydrochemical facies and the geochemical evolution of the groundwater in the Gonbad Plain are effectively illustrated by the Piper diagram (Fig. 8). The distribution of samples on the diagram reveals a complex hydrochemical system dominated by two primary water types: Na-Cl and mixed Na-Ca-HCO₃, with a clear evolutionary pathway from fresh, bicarbonate-rich water to saline, chloride-dominated water. The cation ternary plot on the lower left shows that the majority of samples are heavily shifted towards the Na⁺+K⁺ apex, indicating that sodium is the predominant cation throughout most of the aquifer. This sodium enrichment is a direct consequence of two synergistic processes identified earlier: the dissolution of halite (NaCl) from evaporite deposits and extensive cation exchange, where Ca²⁺ in the groundwater is exchanged for Na⁺ on clay mineral surfaces. The anion ternary plot on the lower right further clarifies the evolutionary trend, showing a wide distribution of samples stretching from the HCO₃⁻ corner to the Cl⁻ corner. This distribution signifies a transition from fresher waters where bicarbonate is the principal anion, typically derived from carbonate weathering in recharge zones, to highly saline waters where chloride, originating from halite dissolution, becomes the dominant anion.

The central diamond field of the Piper diagram synthesizes these ionic characteristics, providing a clear classification of the water types and their controlling mechanisms. A significant portion of the samples falls squarely into the Na-Cl facies (Zone 2), representing the most chemically evolved and saline groundwater in the study area. The dominance of sodium and chloride in these samples is unequivocal evidence that the dissolution of halite, amplified by high evaporation rates, is the terminal process governing the water chemistry in the downgradient parts of the plain. Another major cluster of samples resides in the mixed Na-Ca-HCO₃ facies (Zone 4). This zone represents a critical transitional stage in the groundwater’s evolution. The mixed ionic character suggests that these waters are formed through the mixing of fresh Ca-HCO₃ type waters from recharge areas with the more evolved Na-Cl type waters. Furthermore, the prevalence of samples in this zone, coupled with the high sodium content, strongly supports the interpretation that active cation exchange is occurring, which enriches the water with sodium while depleting calcium. The relative scarcity of samples in the fresh Ca-HCO₃ facies (Zone 1) suggests that most of the groundwater in the sampled wells has already undergone significant geochemical modification and has moved beyond the initial stages of rock-water interaction. The Piper diagram provides a powerful visual confirmation of the hydrogeochemical model for the Gonbad Plain: a system where fresh Ca-HCO₃ waters evolve through mixing and intensive rock-water interactions (cation exchange, silicate weathering) into a mixed Na-Ca-HCO₃ type, and ultimately, under the influence of evaporite dissolution and arid-climate evaporation, into a saline Na-Cl type water unsuitable for many uses.

The Gibbs diagrams (Fig. 8), which plot TDS against the ionic ratios of Na⁺/(Na⁺+Ca²⁺) and Cl⁻/(Cl⁻+HCO₃⁻), offer a macroscopic view of the dominant mechanisms controlling the overall groundwater chemistry. The diagrams clearly show that the vast majority of groundwater samples fall within the Rock-Water Interaction and Evaporation Dominance zones, with only a negligible number (6.7%) showing influence from precipitation dominance. This indicates that the initial chemical signature of the groundwater is overwhelmingly derived from its interaction with the geological formations of the aquifer, rather than being dictated by the chemistry of atmospheric precipitation. The cation diagram (Gibbs I) reveals that 95.6% of the samples have a Na⁺/(Na⁺+Ca²⁺) ratio greater than 0.5, confirming that Na⁺ is the dominant cation across nearly the entire salinity spectrum. This is a direct consequence of the combined effects of halite dissolution and cation exchange. The anion diagram (Gibbs II) is particularly illustrative of the hydrochemical evolution, showing a distinct bimodal distribution. Samples in the Rock-Water Interaction zone (TDS 500–1500 mg/L) exhibit lower Cl⁻/(Cl⁻+HCO₃⁻) ratios, indicating that bicarbonate, derived from carbonate and silicate weathering, is a significant anion in the fresher water. As the TDS increases into the Evaporation Dominance zone (TDS > 1500 mg/L), the Cl⁻/(Cl⁻+HCO₃⁻) ratio shifts dramatically towards 1.0. This trend clearly demonstrates a hydrochemical evolution from a fresher, HCO₃⁻-type water to a highly saline, Cl⁻-dominated water. This evolution is driven by the progressive dissolution of halite and the concentrating effect of high evaporation rates in the semi-arid climate, which removes fresh water and leaves behind dissolved salts. In summary, the Gibbs diagrams confirm that the groundwater chemistry of the Gonbad Plain is controlled by a sequence of processes beginning with rock-water interaction (dissolution of evaporites and weathering of silicates) and is subsequently dominated by evaporation, leading to the high salinity observed in a significant portion of the study area.

The Na⁺ vs. Cl⁻ bivariate plot (Fig. 9) reveals that the majority of samples plot below the 1:1 halite dissolution line, indicating a systematic excess of Na⁺ relative to Cl⁻. While the moderate correlation (R² = 0.85) confirms that halite dissolution contributes to the co-occurrence of these ions, particularly in the low-concentration samples that approach the 1:1 line. it is not the dominant control. The pronounced Na⁺ excess observed in the high-salinity samples implies that additional processes are supplying Na⁺ to the groundwater independently of Cl⁻. Two mechanisms are most plausible in this hydrogeological context: (1) silicate weathering, particularly the hydrolysis of Na-bearing feldspars (e.g., albite), which releases Na⁺ and HCO₃⁻ without a corresponding increase in Cl⁻; and (2) reverse cation exchange, whereby Ca²⁺ in the groundwater is exchanged for Na⁺ adsorbed onto clay minerals in the aquifer matrix, thereby enriching the water in Na⁺.

The Na⁺ vs. (Ca²⁺+Mg²⁺) plot (Fig. 9) reveals a distinctive pattern that provides strong evidence for cation exchange as a dominant hydrochemical process. The most notable feature of this plot is the remarkably narrow and stable range of Ca²⁺+Mg²⁺ concentrations (~ 4–8 meq/L) across nearly all samples, even as Na⁺ increases dramatically from approximately 3 to 33 meq/L. This decoupling where Na⁺ enrichment is not accompanied by a proportional increase in Ca²⁺+Mg²⁺, is the geochemical fingerprint of active direct cation exchange: Ca²⁺ and Mg²⁺ are continuously removed from solution as they are adsorbed onto clay mineral surfaces, while Na⁺ is released into the groundwater. If carbonate or gypsum dissolution were the primary driver of salinity, Ca²⁺+Mg²⁺ would increase proportionally with ionic strength, which is clearly not observed. The majority of samples plot above the 1:1 line, confirming the dominance of this exchange process. One outlier sample, with Ca²⁺+Mg²⁺ ≈ 21 meq/L and relatively low Na⁺, plots below the 1:1 line in the reverse ion exchange domain suggesting that in this localized zone, Na⁺ is being exchanged for Ca²⁺+Mg²⁺, a process associated with evaporative concentration.

This interpretation is reinforced by the (Ca²⁺+Mg²⁺) vs. (HCO₃⁻+SO₄²⁻) plot, where the majority of samples plot above the 1:1 line in the silicate weathering domain. The excess of HCO₃⁻+SO₄²⁻ relative to Ca²⁺+Mg²⁺ is consistent with a scenario in which Ca²⁺ and Mg²⁺ are being depleted by cation exchange faster than they are being supplied by mineral dissolution, while HCO₃⁻ continues to accumulate from carbonate weathering. The two samples falling below the 1:1 line indicate localized carbonate weathering where Ca²⁺+Mg²⁺ production exceeds the anion supply, consistent with the reverse ion exchange sample.

The Na⁺ vs. SO₄²⁻ plot (Fig. 9) shows a moderate correlation (R² = 0.67) and reveals two distinct sample populations: a low-salinity cluster (SO₄²⁻ < 4 meq/L, Na⁺ < 11 meq/L) representing fresher recharge-zone waters, and a high-salinity group (SO₄²⁻ > 5 meq/L, Na⁺ > 20 meq/L) representing more evolved waters from the northern discharge zone. Importantly, this correlation does not imply a direct geochemical link between Na⁺ and SO₄²⁻, as these ions are derived from entirely different source minerals: Na⁺ originates primarily from halite dissolution and cation exchange, while SO₄²⁻ is supplied by gypsum dissolution (CaSO₄·2 H₂O → Ca²⁺ + SO₄²⁻). The apparent co-variation is therefore a spurious correlation driven by the common salinity gradient along the groundwater flow path from south to north, where both ions increase as overall mineralization intensifies. The large scatter in Na⁺ at high SO₄²⁻ values (Na⁺ ranging from ~ 9 to ~ 33 meq/L at SO₄²⁻ > 5 meq/L) further confirms that these two ions are controlled by independent processes operating at different spatial scales within the aquifer.

Collectively, these ionic relationships reveal a clear hydrochemical evolution along the groundwater flow path. The initial mineralization of recharge waters is driven by the dissolution of carbonate and evaporite minerals (calcite, dolomite, halite, and gypsum). As groundwater moves northward through clay-rich aquifer zones, cation exchange becomes the dominant modifying process, progressively enriching the water in Na⁺ while buffering Ca²⁺+Mg²⁺ concentrations within a narrow range. Silicate weathering provides an additional, independent source of Na⁺ and HCO₃⁻, particularly in the deeper flow paths. The result is a groundwater chemistry that evolves from a Ca–Mg–HCO₃ type in the recharge zone to a Na–Cl–SO₄ type in the discharge zone, with cation exchange, rather than simple evaporite dissolution as the primary driver of this evolution.

### Heavy metal pollution index (HPI)

**Fig. 10:**
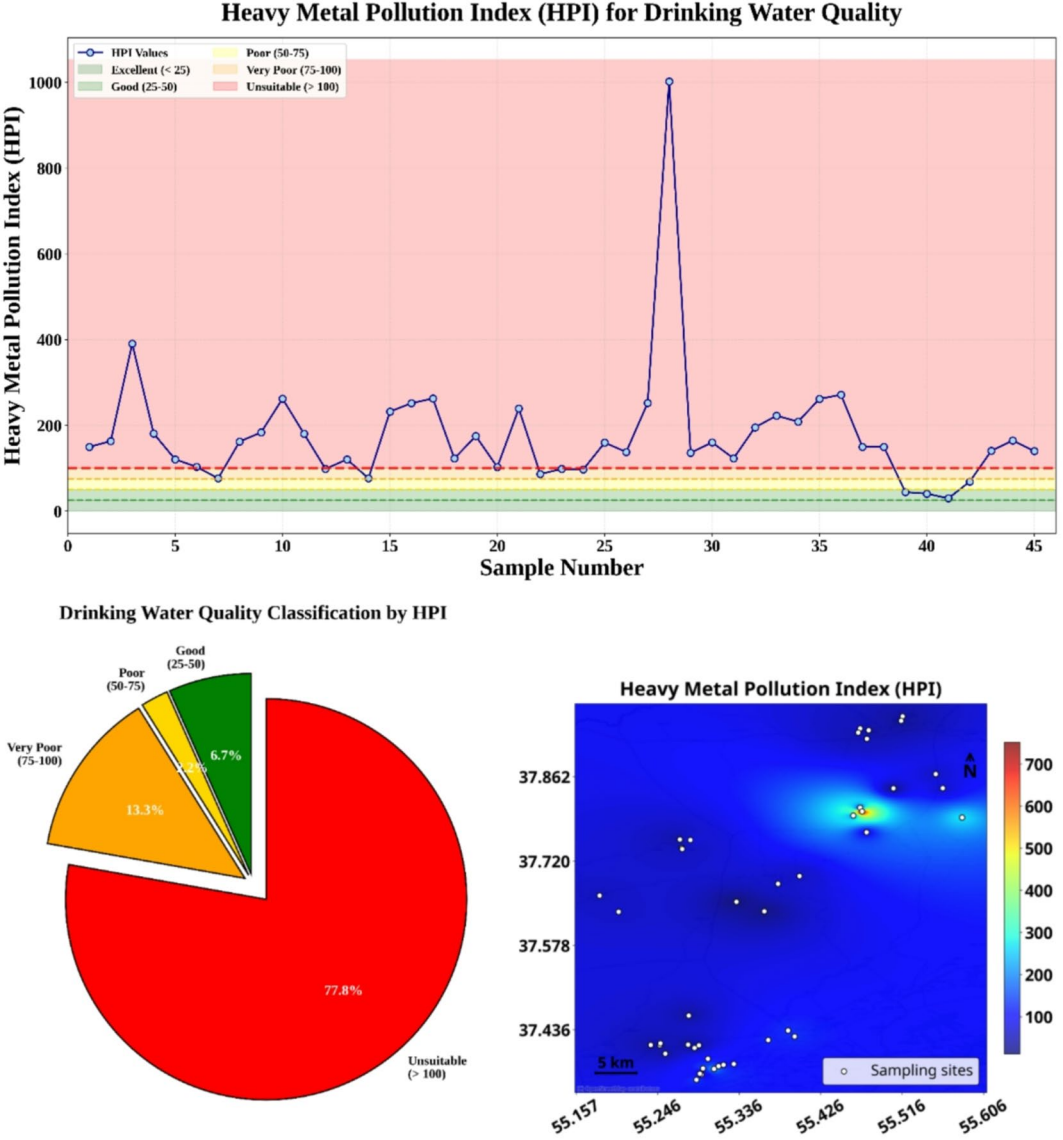
Heavy Metal Pollution Index (HPI) values for the 45 groundwater samples and distribution map of HPI value in the study area. The scatter plot illustrates the HPI for each sample, with the color-coded bars representing the drinking water quality classification: Good (HPI < 50), Poor (50–75), Very Poor (75–100), and Unsuitable for Drinking (HPI > 100). The red dashed line indicates the critical threshold of HPI = 100.

To provide a comprehensive assessment of the overall pollution status of the groundwater with respect to heavy metals, HPI was calculated for each of the 45 samples (Fig. 10). The HPI is a rating method that provides a single, aggregate value representing the total potential risk from the combined effect of various heavy metals. This index is a powerful tool for evaluating the suitability of water for drinking purposes. For this analysis, the groundwater samples were classified according to the standard HPI classification for drinking water quality: Excellent: HPI < 25, Good: 25 ≤ HPI < 50, Poor: 50 ≤ HPI < 75, Very Poor: 75 ≤ HPI ≤ 100, and Unsuitable for Drinking: HPI > 100.

The HPI values for the groundwater samples from the Gonbad Plain reveal a critical water quality situation. The values range from a minimum of 29.62 to an alarming maximum of 1001.91, with a mean HPI of 177.53. This mean value is significantly above the threshold of 100, indicating that the groundwater in the study area is, on average, unsuitable for drinking without treatment. The distribution of the samples across the drinking water quality categories presents a concerning picture: Unsuitable for Drinking (77.8%): The vast majority of the samples, 35 out of 45 (77.8%), have HPI values exceeding 100, rendering them unsuitable for direct consumption as drinking water. This represents a severe public health risk if this water is being used without adequate treatment.

Very Poor Quality (13.3%): An additional 6 samples (13.3%) fall into the “Very Poor” category (75 ≤ HPI ≤ 100). While technically below the unsuitable threshold, water in this range is of marginal quality and poses potential health concerns with prolonged consumption.

Poor Quality (2.2%): Only 1 sample (2.2%) is classified as “Poor” (50 ≤ HPI < 75).

Good Quality (6.7%): A mere 3 samples (6.7%) are classified as “Good” (25 ≤ HPI < 50). These represent the only locations where the groundwater approaches acceptable quality, though it still does not meet the “Excellent” standard.

Two samples stand out as extreme contamination hotspots requiring urgent investigation and remediation: Sample 28: HPI = 1001.91 – This is the most severely contaminated sample, with an HPI value ten times higher than the unsuitable threshold. This represents an acute public health hazard. Sample 3: HPI = 390.57 – This sample also exhibits extremely high contamination, nearly four times the unsuitable threshold. These hotspots suggest the presence of localized, high-intensity sources of heavy metal pollution, which could include industrial discharge points, improper waste disposal sites, or intensive agricultural activities.

The HPI assessment reveals a critical groundwater quality crisis in the Gonbad Plain. With nearly 78% of the samples unsuitable for drinking, the use of untreated groundwater from this aquifer poses a significant and widespread public health risk. The situation is particularly alarming given that groundwater is often the primary source of drinking water in many rural and semi-urban areas. The extremely high HPI values observed in certain samples indicate that the contamination is not uniformly distributed but is concentrated in specific areas. This spatial variability suggests that the sources of heavy metal pollution are localized and may be anthropogenic in origin, such as industrial effluents, agricultural runoff containing pesticides and fertilizers, or leachate from waste disposal sites.

### Health risk assessment based on HQ and HI indices

To evaluate the potential non-carcinogenic health risks associated with the consumption and use of groundwater from the Gonbad Plain, a health risk assessment was conducted. The HQ for individual contaminants (Iron and Manganese) and the HI for combined exposure were calculated for two population groups (adults and children) and two exposure pathways (dermal contact and oral ingestion). A Hazard Index value greater than 1.0 is considered to indicate a potential risk to human health.

In this study, the non-carcinogenic health risk assessment focused specifically on trace metals, namely iron (Fe) and manganese (Mn), due to their elevated concentrations in the aquifer. While nitrate (NO₃⁻) is often a primary concern in agricultural regions, the analytical results demonstrated that nitrate concentrations in the Gonbad Plain ranged from 0.50 to 37.00 mg/L (mean: 8.42 mg/L). Because all samples remained well below the World Health Organization (WHO) permissible limit of 50 mg/L for drinking water, nitrate was excluded from the formal HQ calculations. Conversely, iron concentrations exceeded the WHO threshold of 0.3 mg/L in 43.8% of the samples, necessitating a detailed evaluation of its potential health impacts via oral and dermal exposure pathways for both adults and children.

#### Dermal exposure pathway

**Fig. 11:**
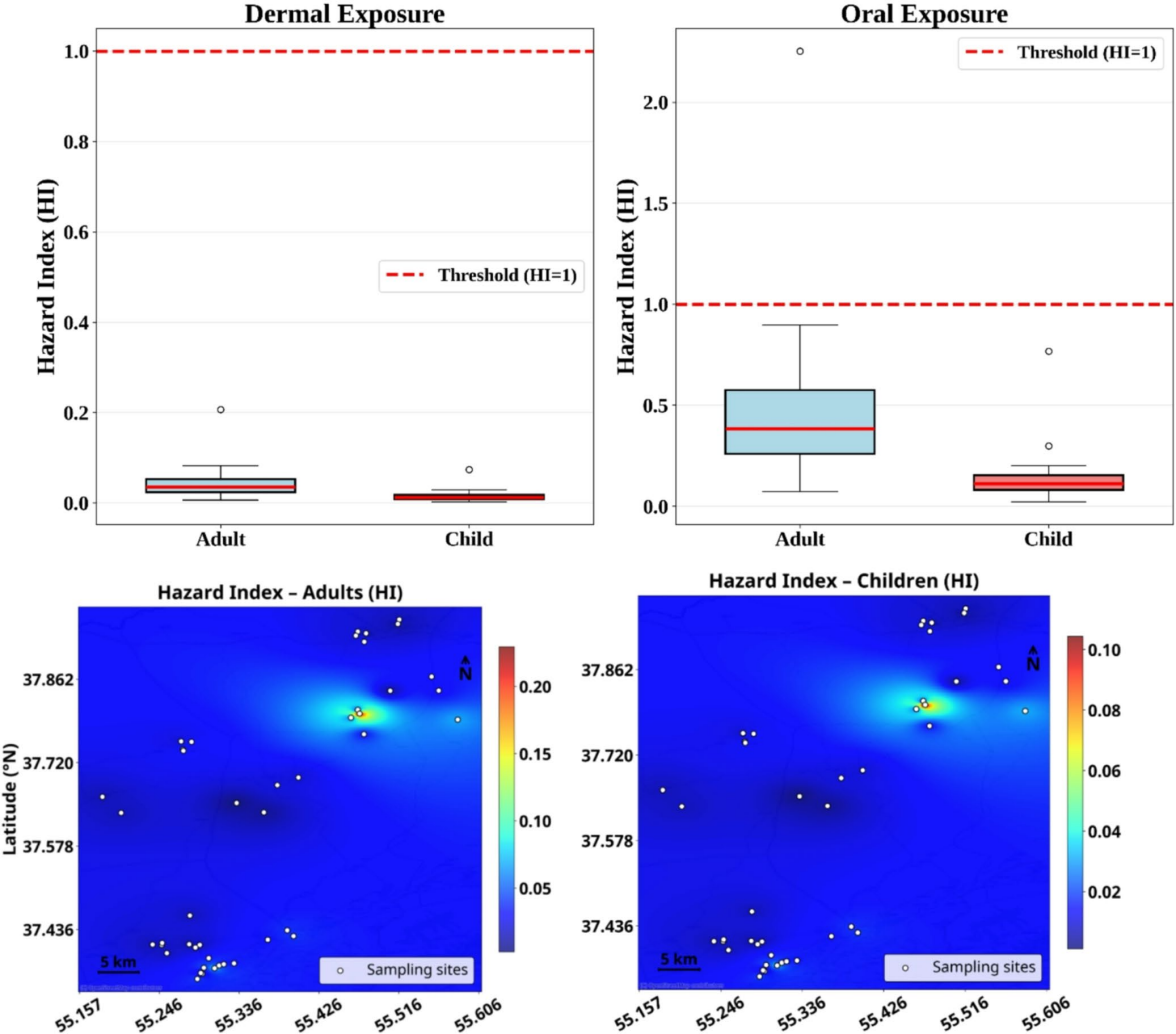
Hazard Index (HI) values calculated for metals in water samples for both populations through oral and dermal contact and distribution map of HI values.

The health risk assessment for the dermal exposure pathway indicates that there is no significant non-carcinogenic risk for either adults or children from contact with the groundwater (Fig. 11). The HI values for both populations were consistently well below the threshold of 1.0 for all 45 samples. For adults, the mean dermal HI was 0.014, with a maximum observed value of 0.074. For children, the mean dermal HI was 0.040, with a maximum value of 0.219.

Although the HI values for children are slightly higher than for adults, which is expected due to their lower body weight and higher skin surface area to volume ratio, they remain in a range that is considered safe. The individual HQ for both Iron (Fe) and Manganese (Mn) through dermal contact were also negligible.

#### Oral exposure pathway

The oral exposure pathway representing the ingestion of groundwater presents a more varied risk profile, particularly for children and in relation to Manganese.

The Hazard Index for the oral pathway reveals a potential health risk in a small subset of the samples, primarily for children (Fig. 11).

For adults, the mean oral HI was 0.134, and in 97.8% of the samples (44 out of 45), the HI was below the safe threshold of 1.0. However, one sample exhibited an HI of 2.93, indicating a potential health risk for adults consuming water from this specific location.

For children, the risk is more pronounced. The mean oral HI was 0.426, which is significantly higher than for adults. While the mean is still below 1.0, the maximum HI value reached 2.93. More importantly, 11.1% of the samples (5 out of 45) had an HI value greater than 1.0 for children, suggesting that the groundwater in these specific locations is not safe for consumption by children.

**Fig. 12:**
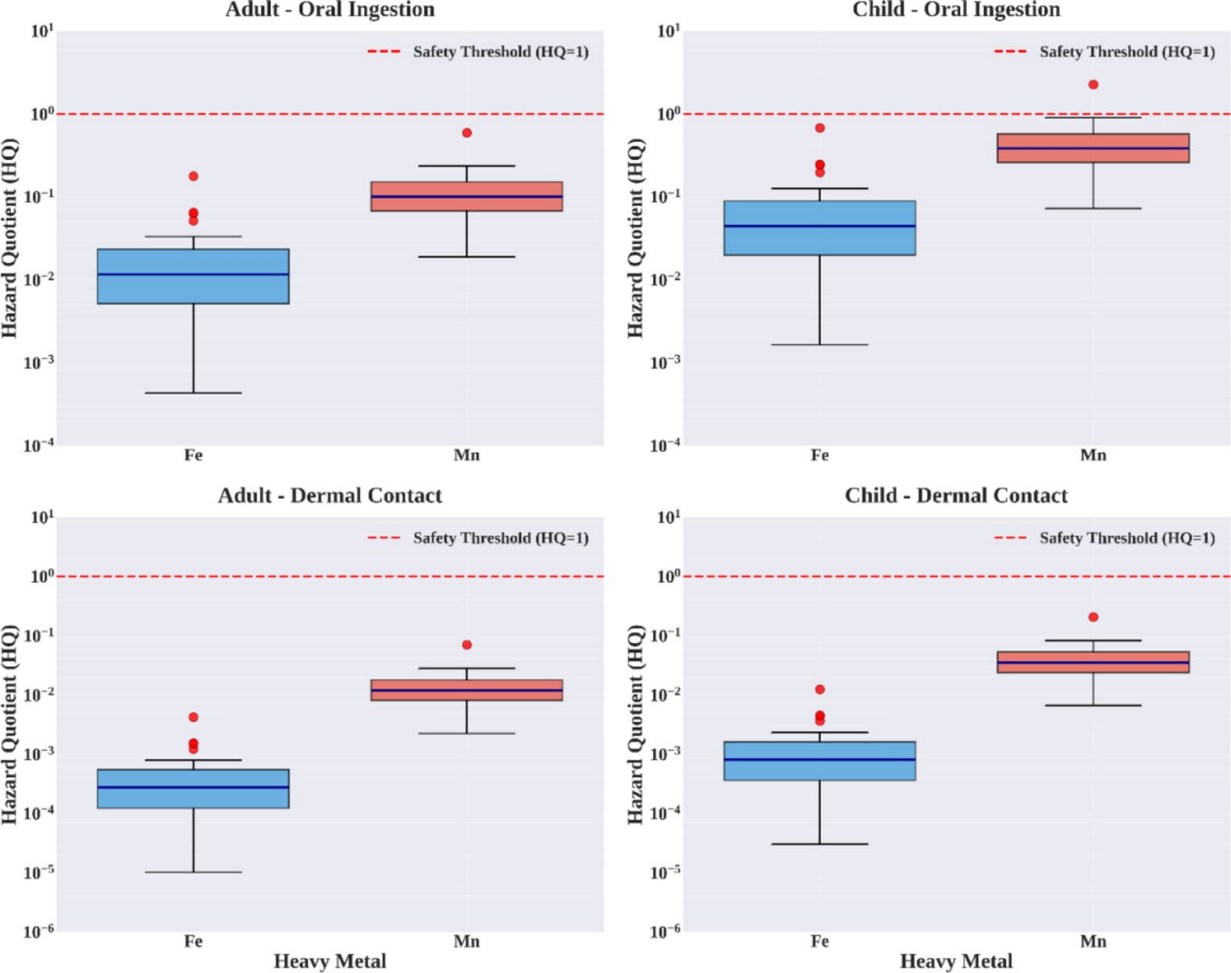
Hazard Quotient (HQ) for non-carcinogenic health risk assessment, plotted on a logarithmic scale. The four-panel box plots show the HQ values for iron (Fe) and manganese (Mn) for both adults and children through oral ingestion and dermal contact pathways. The logarithmic scale enhances the visibility of the wide range of HQ values. The red dashed line at HQ = 1.0 represents the safety threshold.

The potential non-carcinogenic health risks from exposure to iron (Fe) and manganese (Mn) in the groundwater of the Gonbad Plain were quantitatively evaluated using the HQ for two primary exposure pathways (oral ingestion and dermal contact) and two population groups (adults and children). The results, visualized on a logarithmic scale for enhanced clarity (Fig. 12), reveal a complex risk profile where the oral ingestion pathway is the predominant source of concern, and manganese is the primary contaminant driving the potential health risks, particularly for children. The HQ values for the dermal contact pathway were found to be negligible for both Fe and Mn across both age groups, with median values ranging from 1.02 × 10⁻⁵ for Fe in adults to 1.19 × 10⁻² for Mn in children. These values are several orders of magnitude below the safety threshold of 1.0, indicating that dermal absorption of these metals from the groundwater does not pose a significant health threat. In stark contrast, the oral ingestion pathway presents a much higher potential for risk. While the HQ values for Fe through oral ingestion were also low (median of 0.0116 for adults and 0.0444 for children), the values for Mn were substantially higher and approached or exceeded the safety limit in several samples. For adults, the oral HQ for Mn had a median of 0.100 and a maximum of 0.590, remaining below the threshold in all samples. However, the situation is more critical for children, who exhibit a significantly higher vulnerability. The median oral HQ for Mn in children was 0.384, approximately 3.8 times higher than for adults, with a maximum value reaching 2.25. This demonstrates that in at least one location, the exposure to manganese alone is more than double the level considered safe for children, highlighting a direct and significant non-carcinogenic health risk. This heightened vulnerability in children is attributable to their lower body weight and proportionally higher water intake rate, which results in a greater dose per unit of body mass. Although the individual HQ for any single metal did not exceed 1.0 for adults, the elevated HQ values for manganese in children are a major concern and are the primary driver for the overall HI exceeding 1.0 in multiple locations, signaling an unacceptable risk of adverse health effects for the most vulnerable segment of the population. These findings highlight the importance of monitoring groundwater quality in the Gonbad Plain, especially in the areas identified with high Hazard Index values. It is recommended that alternative sources of drinking water or water treatment of the current water resources to be considered for the affected communities, particularly for children, to mitigate the potential health risks associated with high manganese intake.

### Comparison with regional and international groundwater studies

To contextualize the hydrochemical evolution and water quality of the Gonbad Plain aquifer, the findings of this study were compared with recent groundwater assessments conducted in similar arid and semi-arid environments (Table 3). The comparison highlights common salinization mechanisms and shared health risk profiles driven by natural and anthropogenic factors.

In the Algerian Sahara, several studies have documented the pervasive influence of evaporite dissolution on groundwater quality. For instance, in the Hassi Messaoud region^56^ and the Continental Intercalary aquifer of Beni Abbes^57^, groundwater is predominantly of the Na–Cl and Ca–SO₄ types, with salinity driven by the dissolution of halite and gypsum, mirroring the processes observed in the northern sector of the Gonbad Plain. Similarly, in the Adrar region^58^ and the Complex Terminal aquifer of Oued Souf^59^, high mineralization (TDS often exceeding 3000 mg/L) and elevated Na⁺ and Cl⁻ concentrations are the primary causes of poor drinking water quality, as evaluated by the Water Quality Index (WQI). The Entropy-Weighted Water Quality Index (EWQI) applied in the phreatic aquifer of Ouargla^60^ and the further confirms that natural salinization, exacerbated by high evaporation rates, renders the majority of groundwater in these semi-arid basins unsuitable for direct human consumption.

Beyond natural mineralization, anthropogenic contamination, particularly nitrate is a shared concern across these regions. In the semi-arid region of Ain Ouassera^61^ and the Reggane aquifer^62^, nitrate concentrations frequently exceed the WHO limit of 50 mg/L, posing significant non-carcinogenic health risks (HQ > 1) to infants and children. While the Gonbad Plain exhibits lower nitrate levels (mean: 8.42 mg/L) that remain within safe limits, the spatial distribution indicates emerging agricultural impacts in the northern areas, suggesting a trajectory similar to these heavily impacted Algerian basins if fertilizer application is not managed.

The health risks associated with trace elements also show regional parallels. In the deep aquifer of Ouargla city^63^, elevated fluoride concentrations present a severe non-carcinogenic risk, particularly to children. Although fluoride was not the primary focus in the Gonbad Plain, the elevated iron (Fe) concentrations (up to 4.12 mg/L) observed in this study pose analogous localized health risks, driven by reducing conditions in clay-rich aquifer matrices. This phenomenon of trace metal mobilization under specific redox conditions is also documented internationally, such as in the Siwa Oasis of Egypt^64^, where high sulfate and nitrate levels co-occur with trace element enrichment, necessitating advanced remediation strategies like geopolymer adsorption.

Collectively, this comparative analysis underscores that the hydrochemical degradation of the Gonbad Plain aquifer characterized by halite/gypsum dissolution, cation exchange, and localized trace metal enrichment is emblematic of the broader challenges facing groundwater resources in arid and semi-arid regions globally.

**Table 3:** Comparison of groundwater quality and hydrochemical processes across arid and semi-arid regions.

Study Area	Dominant Water Type	Primary Geochemical Processes	Key Contaminants/ WQI Status	Health Risk Findings	Reference
**Gonbad Plain**,** Iran** **(This study)**	Ca–HCO₃ → Na–Cl	Halite/gypsum dissolution, cation exchange, evaporative concentration	High EC, TDS, Fe Future work: WQI	Fe: localized non-carcinogenic risk; NO₃⁻ within safe limits	This study
Hassi Messaoud, Algeria	Na–Cl, Ca–SO₄	Evaporite dissolution, evaporation	High salinity, Cl⁻, SO₄²⁻; WQI: poor	Not assessed	^56^
Beni Abbes, Algeria	Na–Cl, Ca–SO₄	Halite/gypsum dissolution	High TDS; WQI: mostly poor	Not assessed	^57^
Adrar, Algeria	Na–Cl, mixed	Mineral dissolution, evaporation	High TDS; WQI: unsuitable	Not assessed	^58^
Oued Souf, Algeria	Na–Cl, Mg–Ca–SO₄–Cl	Evaporite/carbonate dissolution, cation exchange	High F⁻; EWQI: very poor	High non-carcinogenic risk from F⁻ (children)	^59^
Ouargla (Phreatic), Algeria	Na–Cl, Ca–Mg–SO₄–Cl	Halite/gypsum dissolution, cation exchange	High salinity; WQI: 66.7% non-potable	Not assessed	^60^
Ain Ouassera, Algeria	Mixed Ca–Mg–Cl	Carbonate/sulfate/halite dissolution	High NO₃⁻; WQI: predominantly poor	HQ > 1 for children and adults	^61^
Reggane, Algeria	Na–Cl, Ca–SO₄	Evaporite dissolution, agricultural return flow	High NO₃⁻, salinity; WQI: poor	High NO₃⁻ risk for vulnerable groups	^62^
Ouargla (Deep), Algeria	Na–Cl	Carbonate dissolution, fluorite weathering	High F⁻; EWQI: 70% unfit	High F⁻ risk (children > adults)	^63^
Siwa Oasis, Egypt	Na–HCO₃, Na–Cl	Mineral dissolution, evaporation, salinization	High SO₄²⁻, NO₃⁻, salinity; WQI: poor	High NO₃⁻ and SO₄²⁻ risk for children	^64^

### Limitations and future work

Despite the comprehensive nature of this study, several limitations should be acknowledged. The sampling campaign was conducted during a single dry season, which may not fully capture the temporal variability of groundwater chemistry across wet and dry cycles. The spatial coverage, while representative at the regional scale, may not resolve fine-scale contamination features associated with localized agricultural activities. Furthermore, the water quality assessment relied on the HPI, which evaluates only trace metal contamination and does not account for the contribution of major ions (Na⁺, Cl⁻, SO₄²⁻) and salinity (EC, TDS) to overall drinking water suitability. Similarly, the health risk assessment was restricted to non-carcinogenic risks from Fe and Mn, and did not include carcinogenic risk evaluation for other potentially relevant trace elements.

Future research should prioritize the application of a holistic multi-parameter water quality index such as the Entropy-Weighted Water Quality Index (EWQI) or the Weighted Arithmetic WQI to provide a more complete evaluation of groundwater potability incorporating all physicochemical parameters. Stable isotope analysis (δ¹⁸O, δ²H, ³H) is recommended to constrain recharge sources and groundwater residence times, while a multi-season monitoring programme would capture temporal dynamics and long-term trends driven by climate variability and agricultural intensification. future studies must include other heavy metals (As, Pb, Cd, Cr). Finally, GIS-based aquifer vulnerability mapping integrating remote sensing and hydrochemical data would support the spatial targeting of water resource management interventions in the Gonbad Plain.

## Conclusions

This study integrated hydrochemical analysis, geochemical modeling, multivariate statistics, and health risk assessment to evaluate groundwater quality in the Gonbad Plain, Iran. The principal findings, corresponding to each research objective, are summarized as follows:

- **Hydrochemical characterization**: Groundwater evolves spatially from a Ca–HCO₃ type in the southern recharge zone to a Na–Cl type in the northern discharge zone, driven primarily by halite and gypsum dissolution, cation exchange within the clay-rich alluvial matrix, and evaporative concentration under the semi-arid climate.
- **Geochemical process identification**: Cation exchange is the dominant hydrochemical process modifying groundwater composition, evidenced by the stable and narrow Ca²⁺+Mg²⁺ concentrations despite wide variation in Na⁺. Silicate weathering provides a secondary, independent source of Na⁺ and HCO₃⁻ along deeper flow paths.
- **Multivariate statistical classification**: PCA and HCA delineated three distinct hydrochemical groups, confirming that natural salinization is the most widespread process, while a subset of samples reflects anthropogenic influence from agricultural activities, particularly elevated nitrate concentrations in the northern sector.
- **Drinking water suitability**: A significant proportion of samples exceed WHO guideline values for EC, TDS, Cl⁻, Na⁺, SO₄²⁻, and Fe, rendering the groundwater unsuitable for direct consumption in large parts of the plain without prior treatment.
- **Health risk assessment**: The Heavy Metal Pollution Index (HPI) identified localized heavy metal pollution hotspots in the north-central zone. Non-carcinogenic health risk (Hazard Index) from Fe and Mn via oral and dermal exposure pathways remained below the critical threshold of 1.0 for both adults and children across all sampled locations.

### Management Implications

The findings underscore the urgent need for (1) a spatially targeted groundwater monitoring network focused on the high-salinity northern zone; (2) regulation of agricultural water use and fertilizer application to limit further nitrate and salinity loading; and (3) provision of point-of-use treatment systems or alternative safe water supplies for communities in HPI-exceedance areas.

### Future Research Directions

Isotopic analysis (δ¹⁸O, δ²H, ³H) is recommended to quantify recharge sources and groundwater residence times. Hydrogeological modeling of the aquifer under projected climate and land-use change scenarios would strengthen the predictive basis for long-term water resource management.

## Electronic Supplementary Material

Below is the link to the electronic supplementary material.

Supplementary Material 1

## Data Availability

The data that supports the findings of this study can be obtained from the corresponding author upon reasonable request.
