# Supplementary material for: Hydrochemical and health risk evaluation of the groundwater in Gonbad Kavus area, northeastern Iran
Source: Sci Rep. 2026 May 6;16:20813. doi: 10.1038/s41598-026-51636-2 (PMC13338422; doi:10.1038/s41598-026-51636-2)
Supplement: Supplementary file 1 — Supplementary Material 1 [file 41598_2026_51636_MOESM1_ESM.docx]

**Table 1S. Statistical summary of physicochemical parameters of groundwater samples from the Gonbad Plain (n = 45) with WHO (2017) drinking water guidelines.**

| **Parameter** | **Unit** | **Minimum** | **Maximum** | **Mean** | **Median** | **Std. Dev.** | **CV (%)** | **WHO Limit** | **Samples > WHO n (%)** |
| --- | --- | --- | --- | --- | --- | --- | --- | --- | --- |
| **Temperature** | °C | 8.00 | 26.80 | 21.42 | 24.00 | 4.79 | 22.3 | < 25 | 10 (22.2%) |
| **pH** | – | 7.10 | 7.70 | 7.48 | 7.54 | 0.17 | 2.3 | 6.5–8.5 | 0 (0.0%) |
| **EC** | µS/cm | 760.00 | 4100.00 | 2214.49 | 1580.00 | 1226.47 | 55.4 | 1500 | 24 (53.3%) |
| **TDS** | mg/L | 478.80 | 2583.00 | 1395.13 | 995.40 | 772.68 | 55.4 | 1000 | 22 (48.9%) |
| **Ca²⁺** | mg/L | 61.60 | 160.00 | 91.36 | 96.20 | 19.80 | 21.7 | 200 | 0 (0.0%) |
| **Mg²⁺** | mg/L | 9.60 | 205.00 | 26.47 | 21.60 | 27.92 | 105.5 | 150 | 1 (2.2%) |
| **Na⁺** | mg/L | 62.76 | 762.91 | 319.94 | 198.95 | 239.63 | 74.9 | 200 | 21 (46.7%) |
| **K⁺** | mg/L | 1.17 | 3.12 | 2.37 | 2.40 | 0.49 | 20.5 | 12 | 0 (0.0%) |
| **Cl⁻** | mg/L | 8.00 | 800.00 | 323.64 | 244.00 | 285.02 | 88.1 | 250 | 21 (46.7%) |
| **SO₄²⁻** | mg/L | 64.00 | 510.00 | 222.29 | 150.00 | 148.53 | 66.8 | 250 | 17 (37.8%) |
| **HCO₃⁻** | mg/L | 32.00 | 619.76 | 384.98 | 383.08 | 140.50 | 36.5 | NS | – |
| **CO₃²⁻** | mg/L | 0 | 0 | 0.00 | 0.00 | 0.00 | 0 | NS | – |
| **NO₃⁻** | mg/L | 0.50 | 37.00 | 7.96 | 3.54 | 10.00 | 125.5 | 50 | 0 (0.0%) |
| **SiO₂** | mg/L | 5.00 | 28.42 | 17.99 | 17.40 | 6.55 | 36.4 | NS | – |
| **Fe** | mg/L | 0.01 | 4.12 | 0.46 | 0.27 | 0.66 | 142.7 | 0.3 | 19 (42.2%) |
| **Mn** | mg/L | 0.01 | 0.47 | 0.09 | 0.08 | 0.07 | 77.4 | 0.4 | 1 (2.2%) |

*Notes: SD = Standard Deviation; CV = Coefficient of Variation; WHO = World Health Organization (2017) Guidelines for Drinking-Water Quality; NS = No specific WHO guideline; – = No exceedance applicable. Green cells indicate 0% exceedance; red cells indicate samples exceeding WHO limits.*
